# Association between parenthood and survival among 30,386 patients hospitalized with COVID-19

**DOI:** 10.1371/journal.pone.0346679

**Published:** 2026-04-20

**Authors:** Etienne Audureau, Charline Jean, Richard Layese, Antoine Neuraz, Paul Brasseur, Nadia Oubaya, Laura Polivka, Arsène Mekinian, Olivier Hermine, Julien Rossignol, on behalf of the AP-HP/Universities/INSERM COVID-19 Research Collaboration and AP-HP COVID CDW Initiative

**Affiliations:** 1 Clinical Research Unit (URC) Henri Mondor Hospital, Assistance Publique Hôpitaux de Paris, Créteil, France, C.E.piA. IMRB U.955, Université Paris Est (UPEC), Créteil, France; 2 Department of Biomedical Informatics, Imagine Institute, Hôpital Necker-Enfants Malade, Assistance Publique Hôpitaux de Paris, University of Paris, Paris, France; 3 INSERM U1163, Imagine Institute, Hôpital Necker-Enfants Malade, Assistance Publique Hôpitaux de Paris, University of Paris Cité, Paris, France; Independent Medical Researcher and Writer, UNITED KINGDOM OF GREAT BRITAIN AND NORTHERN IRELAND

## Abstract

**Background:**

Cross-reactive immune responses between endemic human coronaviruses and SARS-CoV-2 have been proposed as a potential factor mitigating COVID-19 severity. Because parents may have more frequent exposure to seasonal respiratory viruses through contact with children, we investigated whether parental status was associated with improved survival among adults hospitalized with COVID-19.

**Methods:**

We conducted a retrospective cohort study using the Assistance Publique–Hôpitaux de Paris (AP-HP) Clinical Data Warehouse. We included all adults (≥18 years) hospitalized with RT-PCR–confirmed SARS-CoV-2 infection between January 29, 2020 and March 1, 2022. The primary endpoint was 90-day mortality after admission, analyzed as a time-to-event outcome using Cox proportional hazards models adjusted for demographics, comorbidities, admission period (pandemic wave), and baseline biological variables. Parental status was extracted from electronic health record text reports. We tested for interaction between age and parental status.

**Results:**

Among 30,386 patients, 22,713 (75%) were classified as having children. Compared with patients without children, those with children were older and had more comorbidities. In the overall adjusted analysis, parental status was not associated with 90-day survival (HR = 1.00, 95%CI 0.94–1.07; p = 0.91). The association between parental status and survival was significantly modified by age (p_interaction_ = 0.002), with an association with lower mortality observed in younger adults that attenuated toward the null in older age groups. After stratification by age (<65 vs ≥ 65 years), having children was associated with improved 90-day survival in patients <65 years (Hazard Ratio (HR)=0.83, 95%CI 0.72–0.95; p = 0.009), whereas no significant association was observed in patients ≥65 years (HR = 1.07, 95%CI 0.99–1.15; p = 0.09).

**Conclusions:**

In this large EHR-based cohort of adults hospitalized with COVID-19, parenthood was associated with lower 90-day mortality among patients younger than 65 years. These findings are observational and may reflect unmeasured confounding; further studies are needed to clarify underlying mechanisms.

## Introduction

The severity of Coronavirus disease 2019 (COVID-19) can vary significantly among individuals. Some people may experience mild symptoms or remain asymptomatic, while others may develop life-threatening complications that require hospitalization and intensive care. Factors such as age and underlying health conditions play a crucial role in the severity of the disease. Several studies have investigated the biological mechanisms involved in COVID-19 severity. Among them, the antiviral response of both adaptive and innate immunity is critical to prevent progression toward a severe form of COVID-19 [1]. Of note, some patients who had never been in contact with SARS-CoV-2 exhibited, *ex vivo*, an immune response against certain epitopes of the virus. This response was related to T- and B-cells cross-immunity between common coronaviruses such as alpha-coronaviruses (HCoV-229E and HCoV-NL63), beta-coronaviruses (HCoV-OC43 and HCoV-HKU1) and SARS-CoV-2 which share common antigens [2]. This cross-immunity has been associated with a decrease in severity of COVID-19, and might be a protective factor against COVID-19 mortality [3–5].

The circulation of respiratory viruses, especially seasonal viruses, is common in paediatric populations [6]. Therefore, workers with children and parents living with children might be more exposed to respiratory viruses, such as endemic coronaviruses, than those not in contact with children. Accordingly, parenthood, associated with living with children and putative contacts with grandchildren depending on the parents’ age, might be linked to a greater likelihood of immunity against endemic coronaviruses and thus increased prevalence of cross-immunity [7].

Several observational studies have examined the association between having children in the household and risk of severe COVID-19 [7–10]. These studies suggest that adults living with children may be at increased risk of SARS-CoV-2 infection, but reduced risk of hospitalization due to SARS-CoV-2 infection. However, data for hospitalized patients on outcomes such as intensive care unit admission and mortality remain limited. To address this gap, we conducted a retrospective study of patients hospitalized for COVID-19, hypothesizing that parents, who have increased contact with children, could have better outcomes during hospitalization than non-parents.

## Materials and methods

### Study design, setting and population

This retrospective cohort study was conducted using data from the Assistance Publique – Hôpitaux de Paris (AP-HP) Healthcare Data Warehouse (HDW), integrating medico-administrative data from standardized discharge reports on diagnoses and procedures performed during the hospital stay as well as information regarding biologic testing and imaging results, drug prescriptions and medical text reports associated with hospital visits.

For the present analysis, included patients were ≥18 years old and hospitalized in one of the AP-HP hospitals because of confirmed SARS-CoV-2 infection from January 29, 2020, to March 1, 2022 (N = 31,963). We excluded patients who died or were discharged within 24 hours after hospital admission (N = 2,466), yielding a total study population of 30,386 patients. During the study period, COVID-19 patients within the AP-HP network (comprising 38 university hospitals) were managed through emergency departments and dedicated COVID-19 wards, with referrals to intensive care units based on clinical severity and bed capacity. To account for temporal shifts in organizational management, therapeutic protocols, and vaccination rollout, all analyses were stratified or adjusted for calendar period (pandemic wave). Additional information on study design, data collection and statistical analysis are given in the **Supplemental materials and methods** section in S1 File.

### Study outcomes

The primary outcome was overall survival at 90 days post-admission. Secondary outcomes included intensive care unit (ICU) admission and in-hospital mortality.

### Data collection

Survival endpoints were collected from EHR data from the AP-HP CDW, in which information on patient mortality is regularly updated from the French National Institute for Statistics and Economic Studies (INSEE). Covariates included demographics, comorbidities identified from electronic health records (EHRs) using International Classification of Diseases, 10th revision, codes (Supplemental Table 1 in S1 File) and biological variables considering the earliest measurement in the 3 days after hospital admission. As the main exposure of interest, parental status was extracted from EHR text reports using regular expressions accounting for typos and spelling errors tailored to identify French words relating to having one or multiple children (“with children”) or explicitly no children (“explicitly without children”); if no mention of children or absence of children was found, patients were classified as “without children”. Patient outcomes including ICU admission, in-hospital death and death at 90 days after admission were collected from the EHRs in the AP-HP HDW, in which information on patient mortality is regularly updated from the French National Institute for Statistics and Economic Studies (INSEE).

### Statistical analyses

No formal sample size calculation was performed; rather, we included the maximum available population to ensure robust estimates. As the primary endpoint of the study, 90-day overall survival was analyzed as a time-to-event endpoint, computing hazard ratios (HRs) by Cox proportional-hazard modeling, along with their corresponding 95% confidence intervals (95% CIs). All analyses were adjusted for a broad selection of prognostic factors, including patient demographics, comorbidities and time period of admission, as detailed in **Supplement**. Because contact with children likely depends on the age of patients and to avoid the limitations of dichotomization, an interaction between age and parental status was evaluated using age as a continuous variable. We compared model fits using linear terms and restricted cubic splines (3–6 knots). Model selection was primarily guided by the Bayesian Information Criterion (BIC) to favour parsimony, as well as the Akaike Information Criterion (AIC) and the Likelihood Ratio Test (LRT). The proportional hazards assumption was assessed through visual inspection of scaled Schoenfeld residual plots. For variables demonstrating deviations from proportionality (notably gender, pandemic wave, and age), a sensitivity analysis was conducted using a stratified Cox model (by gender and wave) with age introduced as a time-dependent coefficient. All analyses were performed after missing data imputation (Supplemental Table 2 in S1 File) using *missForest* [11], a nonparametric method based on random-forest imputation that accommodates non-linearities and interactions. All analyses were performed at the two-tailed P < 0.05 level, using Python 3.7 for data collection and preprocessing, and R 4.4.1 [12] for data imputation and multivariate analyses (R Foundation for Statistical Computing, Vienna, Austria; packages missForest, survival).

The study was conducted in accordance with the Declaration of Helsinki and was approved by the institutional review board (authorization no. IRB 00011591) of the AP-HP Clinical Data Warehouse (CSE 20–11_COVIPREDS).

## Results

### Study population

The main characteristics of the study population are in Table 1 and additional details are in Supplemental Table 3 in S1 File; the distribution of patient hospitalizations over time by COVID-19 wave is in Supplemental Figure 1 in S1 File. Among the 30,386 patients included, parenthood was mentioned in medical records for 22,713 (75%; “with children”), with 7,673 (25%) “without children”. As compared with patients without children, for those with children, the mean age was greater (66.2 ± 17.5 vs. 56.3 ± 21.5; p < 0.0001), number of males lower (54.4% vs. 57.9%, p < 0.0001), median number of comorbidities higher (2.0 vs. 1.0, p < 0.0001), and frequency of risk factors for COVID-19 mortality globally increased, including diabetes (30.2% vs 17.5%, p < 0.0001), obesity (17.0% vs 13.2%, p < 0.0001), and congestive heart failure (18.4% vs 11.5% p < 0.0001). Overall, day-28 and −90 unadjusted survival rates were increased for patients with children (p < 0.0001, Table 1), along with hospital mortality, ICU admission and length of stay.

**Table 1:** Main characteristics of patients with COVID-19, without or with children over the study period.

Characteristic	OverallN = 30386^1^	Without childrenN = 7673^1^	With childrenN = 22713^1^	p-value^2^
**Demographics**				
Age, years	63.7 ± 19.1	56.3 ± 21.5	66.2 ± 17.5	**<0.001**
Male sex	16,801 (55.3%)	4,442 (57.9%)	12,359 (54.4%)	**<0.001**
**Outcomes**				
28-day mortality	4,572 (15.0%)	892 (11.6%)	3,680 (16.2%)	**<0.001**
90-day mortality	5,711 (18.8%)	1,152 (15.0%)	4,559 (20.1%)	**<0.001**
In hospital mortality	4,917 (16.2%)	995 (13.0%)	3,922 (17.3%)	**<0.001**
ICU transfer	8,960 (29.5%)	2,115 (27.6%)	6,845 (30.1%)	**<0.001**
Length of hospital stay, days	9.0 [5.0;16.0]	8.0 [4.00;15.0]	9.0 [5.0;17.0]	**<0.001**
**Comorbidities**				
Number of comorbidities	2.00 [1.00;4.00]	1.00 [0.00;3.00]	2.00 [1.00;4.00]	**<0.001**
Diabetes	8,199 (27.0%)	1,346 (17.5%)	6,853 (30.2%)	**<0.001**
Obesity	4,876 (16.0%)	1,013 (13.2%)	3,863 (17.0%)	**<0.001**
Body mass index, kg/m²	27.2 ± 4.23	27.0 ± 4.28	27.3 ± 4.21	**<0.001**
Hypertension	12,986 (42.7%)	2,316 (30.2%)	10,670 (47.0%)	**<0.001**
Congestive heart failure	5,073 (16.7%)	885 (11.5%)	4,188 (18.4%)	**<0.001**
Cardiac arrythmia	6,024 (19.8%)	1,131 (14.7%)	4,893 (21.5%)	**<0.001**
Chronic pulmonary disease	3,927 (12.9%)	833 (10.9%)	3,094 (13.6%)	**<0.001**
Renal failure	4,815 (15.8%)	845 (11.0%)	3,970 (17.5%)	**<0.001**
Dementia	3,944 (13.0%)	775 (10.1%)	3,169 (14.0%)	**<0.001**
Solid tumor without metastasis	3,085 (10.2%)	612 (8.0%)	2,473 (10.9%)	**<0.001**
Metastatic cancer	1,256 (4.1%)	235 (3.1%)	1,021 (4.5%)	**<0.001**
**Biologic variables**				
Na, mmol/L	137 [134;139]	137 [134;139]	136 [134;139]	**<0.001**
K, mmol/L	4.06 [3.78;4.40]	4.00 [3.77;4.30]	4.09 [3.79;4.40]	**<0.001**
Hemoglobin, g/dL	12.8 [11.6;14.1]	13.1 [11.8;14.3]	12.8 [11.6;14.0]	**<0.001**
Leukocyte count, x 10^9 / L	7.0 [5.2;9.2]	7.3 [5.4;9.7]	7.0 [5.2;9.1]	**<0.001**
Lymphocyte count, x 10^9 / L	1.03 [0.70;1.41]	1.07 [0.73;1.51]	1.01 [0.70;1.38]	**<0.001**
Platelet count, x 10^9 / L	219 [170;269]	224 [176;274]	217 [168;267]	**<0.001**
Serum creatinine, µmol/L	79.0 [62.0;107]	75.7 [60.0;98.0]	81.0 [63.0;109]	**<0.001**
Urea, mmol/L	6.1 [4.30;9.3]	5.5 [3.80;8.4]	6.3 [4.50;9.6]	**<0.001**
C-reactive protein (mg/L)	59.8 [20.0;121]	49.6 [15.0;109]	63.0 [22.6;125]	**<0.001**
Protides, g/L	71.0 [66.8;75.0]	71.3 [67.0;75.6]	71.0 [66.3;75.0]	**<0.001**
Lactate dehydrogenase, U/L	363 [280;474]	352 [266;472]	366 [283;474]	**<0.001**
D-Dimer (µg/L)	1,107 [744;1,674]	1,090 [712;1,673]	1,113 [756;1,674]	**0.008**
Arterial blood gas test: SpO2, %	94.4 [92.3;95.8]	94.5 [92.6;95.9]	94.4 [92.2;95.8]	**<0.001**
Arterial blood gas test: PaO2, mmHg	73.3 [63.1;81.3]	74.1 [64.6;82.3]	73.1 [62.7;81.0]	**<0.001**
Arterial blood gas test: PaCO2, mmHg	35.2 [32.1;38.1]	35.2 [32.2;38.1]	35.2 [32.0;38.1]	0.54

^1^Mean ±SD; n (%); median [25%;75%]

^2^Welch two sample t-test; Pearson#39;s chi-squared test; Wilcoxon rank sum test, ICU, intensive care unit

### Survival analyses

Main results from adjusted 90-day OS analyses are in **Table 2**, globally and by age class (median <65/ ≥ 65 years), with detailed results including all covariables in Supplemental Tables 4-6 in S1 File.

**Table 2:** Univariate and multivariate Cox proportional-hazards modeling for 90-day overall survival: results for patients with COVID-19, without or with children and by age group.

	Univariable analysis		Multivariable analysis	
Characteristic	HR (95% CI)^1^	p-value	HR (95% CI)^2^	p-value
Whole study population				
Without children	—		—	
With children	1.38 (1.29;1.47)	**<0.001**	1.004 (0.94;1.07)	0.91
Patients <65 years old				
Without children	—		—	
With children	1.27 (1.12;1.45)	**<0.001**	0.83 (0.72;0.95)	**0.009**
Patients ≥65 years old				
Without children	—		—	
With children	1.03 (0.95;1.11)	0.49	1.07 (0.99;1.15)	0.088

^1^Hazard ratio (95% confidence interval) from unadjusted Cox proportional-hazards regression modeling.

^2^Hazard ratio (95% confidence interval) from multivariable Cox proportional-hazards regression modeling adjusted for demographics, comorbidities and biological variables at baseline (detailed in Supplemental material in S1 File).

**Fig 1:**
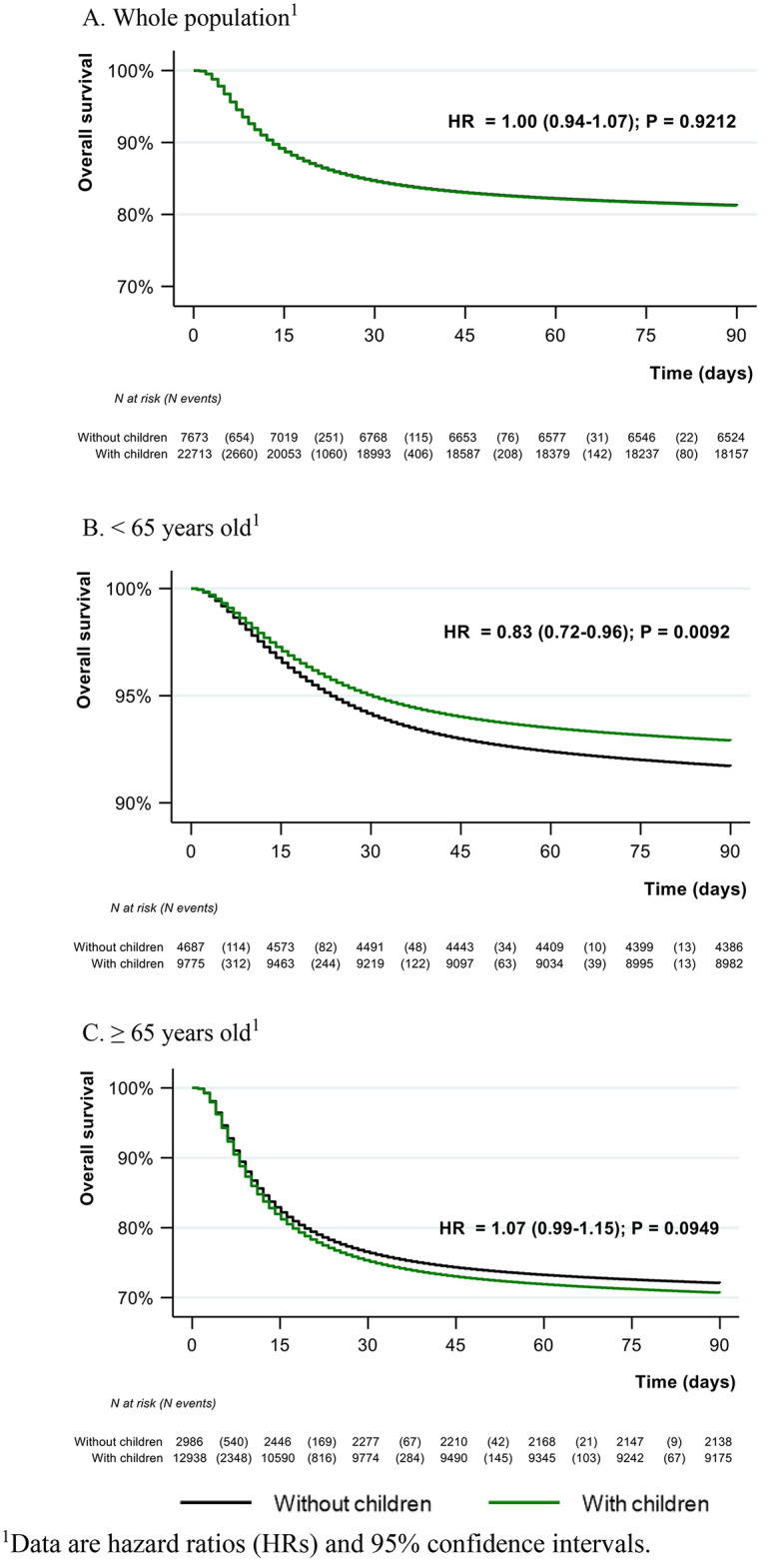
Day-90 adjusted overall survival curves by parental status and by age category.

First, considering global results regardless of age, adjusted OS did not significantly differ between the 2 groups, considering the whole period of inclusion (HR = 1.00 [95% CI 0.94;1.07], p = 0.91; **Fig 1A** and Supplemental Figure 2 in S1 File) or each individual COVID-19 wave (Supplemental Figures 3-7 in S1 File).

A formal interaction analysis was then conducted to assess whether the association between parental status and overall survival was significantly modified by age. Based on the BIC, a linear interaction model between parental status and age as a continuous variable was the most parsimonious, while a 5-knot spline model provided an alternative fit using AIC and LRT. Both approaches found a significant interaction (p = 0.002 for the linear form, p = 0.022 for the 5-knot spline form), confirming that the association between parental status and 90-day mortality is significantly modified by age. Considering the linear interaction model, parental status conferred a substantial survival advantage in younger ages (HR_’with children’_ at the intercept = 0.6019 [0.4361; 0.8307], p = 0.002), which progressively diminished by a factor of 1.0069 per additional year of age (HR_age*’with children’_ = 1.0069 [95% CI 1.0026;1.0112], p = 0.002; HR_age_ = 1.0538 [1.0497; 1.0579], p < 0.001). Visual inspection of the interaction plots (Supplemental Figures 8-9 in S1 File) shows that the association is robust in younger adults and gradually moves toward the null in older populations. To facilitate the interpretation of the identified interaction, subsequent survival analyses were stratified using a median-split (<65 vs. ≥ 65 years). For patients <65 years old, we found an association between having children and overall day-90 survival (HR = 0.83 [95% CI 0.72;0.96], p = 0.009), with no significant association found for patients ≥65 years (HR = 1.07 [95% CI 0.99;1.15], p = 0.09; Figs 1B,C).

### Sensitivity analyses

A sensitivity analysis discarding variables with missing data >30% (16 variables excluded, namely body mass index, glycemia, HCO3- serum, calcium, fibrinogen, procalcitonin, troponin, LDH, creatine kinase, D-dimer, BGA: oxygen saturation, BGA: PaO2, BGA: PaCO2, BGA: HCO3-, BGA: lactate, BGA: pH) produced similar results (whole population: adjusted HR = 1.003 [95% CI 0.94;1.07], p = 0.94; < 65y: adjusted HR = 0.85 [95% CI 0.74;0.98], p = 0.024; ≥ 65 years old: adjusted HR = 1.05 [95% CI 0.97;1.13], p = 0.22).

Sensitivity analyses using log-transformed values for highly skewed variables (including creatine kinase, troponin, lactate dehydrogenase, procalcitonin, total bilirubin, ALT, CRP, prothrombin time, serum creatinine, and urea) yielded robust results consistent with the primary analysis. Specifically, the associations for parental status remained stable: in the whole population (HR 1.019; [95% CI 0.954;1.088], p = 0.58), in the < 65 years subgroup (HR 0.857; [95% CI 0.746;0.985], p = 0.030), and in the ≥ 65 years subgroup (HR 1.078; [95% CI 0.999;1.163], p = 0.052).

Proportional hazards diagnostics and scaled Schoenfeld residual plots are provided in Supplemental Figures 10 and 11 in S1 File. A sensitivity analysis accounting for non-proportionality of gender, wave, and age confirmed the stability of our primary estimates, particularly the association between parental status and lower mortality in patients <65 years (whole population: adjusted HR = 1.006 [95% CI 0.941;1.074], p = 0.867; < 65 years: adjusted HR = 0.834 [0.726;0.959], p = 0.011; ≥ 65 years old: adjusted HR = 1.069 [0.991;1.154], p = 0.085).

To address potential misclassification within the ‘no mention’ group, a sensitivity analysis was performed using a three-level categorical variable for parental status (Explicitly childless, Explicitly with children, and No mention). The ‘no mention’ group was markedly younger (mean age 50.1 years ±20.4, vs. 63.5 ± 20.3 [explicitly without children] and 66.2 ± 17.5 [explicitly with children]) and had fewer comorbidities than the other two groups, suggesting a different clinical profile or less comprehensive documentation for younger, less severe cases (Supplemental Table 7 in S1 File). To account for the age disparity between groups, we performed an age-standardization of baseline characteristics and outcomes (Supplemental Table 8 in S1 File). Upon standardization, the prevalence of major comorbidities (e.g., hypertension, arrythmia and congestive heart failure) was found to be broadly similar between those explicitly identified as parents and those identified as childless. The ‘no mention’ group remained characterized by a lower burden of comorbidities but exhibited a slightly higher age-standardized 90-day mortality rate compared to the other two groups. After multivariable adjustment, the association between parenthood and lower 90-day mortality in patients <65 years remained significant (Supplemental Table 9 in S1 File). Compared to the explicitly childless group (Reference, HR = 1.0), patients explicitly identified as parents had a lower risk of 90-day mortality (HR 0.81; 95% CI 0.68–0.97, p = 0.024), whereas no significant difference was observed for the ‘no mention’ group (HR 0.95; 95% CI 0.76–1.20, p = 0.68). Finally, to assess the potential for overadjustment bias, we performed a sequential multivariable analysis, by [Model 1] adjusting for demographics only (Age, Gender), [Model 2] further adjusting for comorbidities (baseline health status), and [Model 3] further adjusting for biomarkers at admission (clinical severity). Adjusting for demographics and comorbidities was sufficient to produce results of similar magnitude and significance to the full model (Model 3), with the subsequent inclusion of laboratory biomarkers only slightly refining the point estimates, suggesting that the primary association is robust to the choice of clinical adjustment variables (Supplemental Tables 10-12 in S1 File).

## Discussion

Prior research has indicated a protective association between parity (particularly at moderate levels) and all-cause mortality [13]. However, studies directly examining the potential protective associations between parenthood and life-threatening viral infections in hospitalized patients remain scarce. This large retrospective study of hospitalized COVID-19 patients shows that being a parent is associated with reduced 90-day mortality in those under 65 years old. We observed a significant attenuation of the hazard ratio after adjusting for baseline characteristics, moving from an unadjusted HR around 1.38 to a near-null association in the whole population and an association with lower mortality in younger patients. This shift is characteristic of strong negative confounding, primarily driven by age. Given that age is the dominant risk factor for COVID-19 mortality, multivariable adjustment was essential to unmask the independent association between parental status and survival.

These findings contribute to the growing body of literature suggesting that, although the presence of children in a household may increase the risk of infection due to higher household exposure to SARS-CoV-2, parental status may be associated with better outcomes during hospitalization. These results suggest that household dynamics and pre-existing immunity may play a role in disease outcomes. From a clinical and policy perspective, these findings highlight the importance of incorporating household-level social determinants into risk stratification models. Of note, we observed a significant interaction between age and parental status: the survival advantage was most pronounced in younger cohorts and attenuated progressively with increasing age. This suggests that the underlying protective mechanisms (potentially linked to frequent exposure to endemic coronaviruses) may diminish or be offset by other age-related physiological factors in older populations.

Several potential mechanisms could explain this observation. One hypothesis is that parenthood may confer a greater probability of cross-immunity to seasonal coronaviruses through frequent exposure to children. In our study, the association between lower mortality and parenthood was observed only in patients younger than 65 years, which may support the hypothesis that frequent exposure to children contributes to improved immune responses. Another consideration is immune senescence, which can impair the ability of the immune system in older adults to respond effectively to infections and provide complete protection following vaccination. From a clinical perspective, this age-dependent pattern suggests that parental status may be more relevant in populations with lower baseline risk, where immunological or behavioral factors could have a greater relative impact, whereas in older patients, outcomes are likely driven predominantly by age-related vulnerability and comorbidities.

While the cross-immunity hypothesis remains a plausible biological explanation for the survival advantage observed in parents, it was not directly tested in our study and must be weighed against alternative mechanisms. For instance, social and behavioral determinants may contribute significantly; parents may benefit from stronger social support networks or exhibit different healthcare-seeking behaviors (such as earlier presentation to the hospital) compared to childless individuals. Furthermore, despite our multivariable adjustment, unmeasured socioeconomic factors and residual confounding inherent to EHR data may persist. In particular, socioeconomic status, household composition, and healthcare-seeking behavior may differ systematically between parents and non-parents and could partially explain the observed association. Indeed, variations in household structure often correlate with lifestyle factors and healthcare access that were not fully captured in our clinical dataset. Consequently, the observed association likely reflects a complex interplay between immunological history and these broader psychosocial determinants.

The primary strengths of this study include the large, multi-center sample size encompassing nearly 40 university hospitals and the implementation of rigorous multivariable models. These models specifically accounted for the modifying effect of age and were supported by a suite of sensitivity analyses that demonstrated the stability of our findings. However, several limitations must be acknowledged. The retrospective and observational nature of the study means that we can only identify associations and cannot establish causality. Future studies incorporating immunological analyses, particularly to assess the potential presence of cross-immunity in parents versus non-parents, are needed to strengthen these findings. To account for this, we conducted multivariable analyses incorporating a wide range of potential confounders. Another significant limitation of our study is the absence of individual-level vaccination status. Given that vaccination significantly reduces COVID-19 mortality and coverage varies by age and household structure, it represents a potential source of residual confounding. In France, vaccination campaigns prioritized older and high-risk individuals starting in early 2021. If the parental survival advantage were driven by higher vaccination rates among parents, the association should logically be absent in the early stages of the pandemic. However, our results demonstrate a consistent association with lower mortality in patients under 65 years during Waves 1 and 2, a period before vaccines were available. This suggests that the observed parental benefit is unlikely to be driven by vaccination status. While adjusting for ‘pandemic wave’ partially captures broad shifts in population immunity and clinical management, the lack of individual data remains a limitation that future prospective studies should address. Nevertheless, residual confounding related to differential vaccination uptake between parents and non-parents, particularly in later waves, cannot be fully excluded and should be considered when interpreting our findings.

Furthermore, the parental status variable was not directly available and was derived from unstructured EHR data using natural language processing (NLP) rather than prospective self-reporting. While our validation of a random subset of records demonstrated excellent positive predictive value, the reliance on clinical notes introduces a potential risk of misclassification bias and omissions, as parental status may not have been systematically documented for all patients. In particular, patients with no explicit mention of parental status may have been misclassified as not having children. However, sensitivity analyses using a three-category definition of parental status yielded consistent results, supporting the robustness of our findings despite this potential misclassification. In addition, the analysis was restricted to hospitalized patients, which may introduce selection bias. Hospital admission is influenced by both disease severity and healthcare-seeking behavior, and these factors may differ according to parental status. Conditioning on hospitalization may therefore act as a collider, potentially biasing the association between parental status and COVID-19 severity. As a result, our findings should be interpreted cautiously and may not reflect the relationship between parental status and disease severity in the broader population of individuals infected with SARS-CoV-2. The direction and magnitude of this bias are difficult to predict, and future studies using population-based cohorts including non-hospitalized cases would be needed to address this limitation frequent in hospital-based COVID studies. Finally, several clinical biomarkers, such as LDH, troponin, and procalcitonin, exhibited high rates of missingness (exceeding 50%). While we addressed this using the missForest imputation algorithm, we acknowledge that the ‘Missing at Random#39; (MAR) assumption may not fully hold in this context, as the decision to measure these markers is often clinical-severity dependent (Missing Not At Random, MNAR). Although we adjusted for a wide range of available clinical covariates to mitigate this and a sensitivity analysis leaving out variables with missing data > 30% confirmed the main results, we cannot entirely rule out the potential for ascertainment bias or residual confounding regarding the influence of these biomarkers on the observed associations.

## Conclusion

In conclusion, our study identifies a significant association between parenthood and lower mortality in younger COVID-19 patients. While these findings suggest that cross-reactive immunity may influence COVID-19 outcomes, the observed association between parental status and survival may also reflect broader socioeconomic and behavioral factors. Future research, particularly incorporating immunological profiling and more detailed characterization of social determinants, is necessary to elucidate the mechanisms underlying this relationship.

## Supporting information

S1 File Supplemental material including the list of members of the AP-HP COVID Clinical Data Warehouse (CDW) Initiative, supplemental materials and methods, Tables S1–S12, and Figures S1–S11. (DOCX)
