# Supplementary material for: Association between parenthood and survival among 30,386 patients hospitalized with COVID-19
Source: PLoS One. 2026 Apr 20;21(4):e0346679. doi: 10.1371/journal.pone.0346679 (PMC13094966; doi:10.1371/journal.pone.0346679)
Supplement: S1 File — (DOCX) [file pone.0346679.s001.docx]

**Supplemental material**

**List of members from the AP-HP COVID Clinical Data Warehouse (CDW) Initiative**

**Supplemental material and methods**

**Supplemental results**

- **Supplemental Table 1.** List of ICD-10 codes used to extract patient comorbidities
- **Supplemental Table 2.** Descriptive statistics of variables with missing information, before-after missing data imputation
- **Supplemental Table 3.** Detailed characteristics of patients with COVID-19 and without and with children over the whole study period
- **Supplemental Table 4.** Results from univariate and multivariate Cox proportional-hazards modeling for 90-day overall survival: whole study population
- **Supplemental Table 5.** Results from univariate and multivariate Cox proportional-hazards modeling for 90-day overall survival: patients aged < 65 years old
- **Supplemental Table 6.** Results from univariate and multivariate Cox proportional-hazards modeling for 90-day overall survival: patients ≥ 65 years old
- **Supplemental Table 7.** Main characteristics of the study population according to parental status (3 categories)
- **Supplemental Table 8.** Age-standardized main baseline characteristics of the study population, stratified by parental status
- **Supplemental Table 9.** Results from univariate and multivariable Cox proportional hazards modeling for 90-day overall survival: results according to parental status as a 3-categories variable
- **Supplemental Table 10.** Sequential multivariable Cox proportional hazards modeling for 90-day overall survival: whole population
- **Supplemental Table 11.** Sequential multivariable Cox proportional hazards modeling for 90-day overall survival: <65y
- **Supplemental Table 12.** Sequential multivariable Cox proportional hazards modeling for 90-day overall survival: >65y
- **Supplemental Figure 1.** Distribution of all hospitalized patients by COVID-19 wave over the whole study period (waves 1 to 5)
- **Supplemental Figure 2.** Day-90 overall survival by parental status over the whole study period
- **Supplemental Figure 3.** Day-90 overall survival by parental status over the 1st COVID-19 wave
- **Supplemental Figure 4.** Day-90 overall survival by parental status over the 2nd COVID-19 wave
- **Supplemental Figure 5.** Day-90 overall survival by parental status over the 3rd COVID-19 wave
- **Supplemental Figure 6.** Day-90 overall survival by parental status over the 4th COVID-19 wave
- **Supplemental Figure 7.** Day-90 overall survival by parental status over the 5th COVID-19 wave
- S**upplemental Figure 8.** Parental status association with 90-day overall survival according to age (linear)
- S**upplemental Figure 9.** Parental status association with 90-day overall survival according to age (5-knots restricted cubic spline)
- S**upplemental Figure 10.** Scaled Schoenfeld residuals, part 1
- S**upplemental Figure 11.** Scaled Schoenfeld residuals, part 2

**List of members from the AP-HP COVID Clinical Data Warehouse (CDW) Initiative**

Pierre-Yves Ancel (APHP Paris University Center), Alain Bauchet (APHP Saclay University), Nathanael Beeker (APHP Paris University Center), Vincent Benoit (WIND Department APHP Greater Paris University Hospital), Romain Bey (WIND Department APHP Greater Paris University Hos- pital), Aurélie Bourmaud (APHP Paris University North), Stéphane Bréant (WIND Department APHP Greater Paris University Hospital), Anita Burgun (Department of Biomedical Informatics, HEGP, APHP Greater Paris University Hospital), Fabrice Carrat (APHP Sorbonne University), Charlotte Caucheteux (Université Paris-Saclay, Inria, CEA), Julien Champ (INRIA Sophia-Antipolis – ZENITH team, LIRMM, Montpellier, France), Sylvie Cormont (WIND Department APHP Greater Paris University Hospital), Julien Dubiel (WIND Department APHP Greater Paris University Hospital), Catherine Duclos (APHP Paris Seine Saint Denis Universitary Hospital), Loic Esteve (SED/SIERRA, Inria Centre de Paris), Marie Frank (APHP Saclay University), Nicolas Garcelon (Imagine Institute), Alexandre Gramfort (Université Paris-Saclay, Inria, CEA), Nicolas Griffon ("WIND Depart- ment APHP Greater Paris University Hospital UMRS1142 INSERM"), Olivier Grisel (Université Paris-Saclay, Inria, CEA), Martin Guilbaud (WIND Department APHP Greater Paris University Hospital), Claire Hassen-Khodja (Direction of the Clini- cal Research and Innovation, AP-HP), François Hemery (APHP Henri Mondor University Hospital), Martin Hilka (WIND Department APHP Greater Paris University Hospital), Anne Sophie Jannot (Department of Biomedical Informat- ics, HEGP, APHP Greater Paris University Hospital), Jerome Lambert (APHP Paris University North), Richard Layese (APHP Henri Mondor University Hospital), Léo Lebouter (WIND Department APHP Greater Paris University Hospital), Damien Leprovost (Clevy.io), Ivan Lerner (Department of Biomedical Informat- ics, HEGP, APHP Greater Paris University Hospital), Kankoe Levi Sallah (APHP Paris University North), Aurélien Maire (WIND Department APHP Greater Paris University Hospital), Marie-France Mamzer (President of the AP-HP IRB), Patricia Martel (APHP Saclay University), Arthur Mensch (ENS, PSL University), Thomas Moreau (Université Paris-Saclay, Inria, CEA), Antoine Neuraz (Department of Biomedical Informatics, HEGP, APHP Greater Paris University Hospital), Nina Or- lova (WIND Department APHP Greater Paris University Hospital), Nicolas Paris (WIND Department APHP Greater Paris University Hospital), Bastien Rance (Department of Biomedical Informatics, HEGP, APHP Greater Paris University Hospital), Hélène Ravera (WIND Department APHP Greater Paris University Hospital), Antoine Rozes (APHP Sorbonne University), Pierre Rufat (APHP Sorbonne University), Elisa Salamanca (WIND Department APHP Greater Paris University Hospital), Arnaud Sandrin (WIND Department APHP Greater Paris University Hospital), Patricia Serre (WIND Department APHP Greater Paris University Hospital), Xavier Tannier (Sorbonne University), Jean-Marc Treluyer (APHP Paris University Center), Damien Van Gysel (APHP Paris University North), Gael Varoquaux (Université Paris-Saclay, Inria, CEA, Montréal Neurologi- cal Institute, McGill University), Jill-Jênn Vie (SequeL, Inria Lille), Maxime Wack (Department of Biomedical Informatics, HEGP, APHP Greater Paris University Hospital), Perceval Wajsburt (Sorbonne University), Demian Wassermann (Université Paris-Saclay, Inria, CEA), Eric Zapletal (Department of Biomedical Informatics, HEGP, APHP Greater Paris University Hospital.

**Supplemental materials and methods**

**Setting and study population**

This retrospective cohort study was conducted using the Assistance Publique-Hôpitaux de Paris (AP-HP) Clinical Data Warehouse (CDW; “Entrepôt de Données de Santé” https://eds.aphp.fr/)(Chouchana, Beeker et al. 2021, Lombardi, Azoyan et al. 2021). This data warehouse contains electronic health records for all inpatients from the 38 greater Paris university hospitals (representing > 11 million patients). It integrates medico-administrative data from the medical information system program (PMSI) and the French national hospital database, which gathers information from standardized discharge reports on diagnoses and procedures performed in all medical units involved in patient management during the hospital stay. Primary and associated diagnoses are recorded using the International Classification of Diseases, 10^th^ revision (ICD-10). In addition to PMSI data, the HDW gathers information from multiple databases, including biologic and imaging results, drug prescriptions (stored within a dedicated medication database and coded according to the international Anatomical Therapeutic Chemical classification system) and medical text reports associated with hospital visits, including emergency department data and outpatient visits.

Our study population included all patients ≥ 18 years old and hospitalized in one of the AP-HP hospitals related to a confirmed SARS-CoV-2 infection from January 29, 2020, to March 1, 2022 (N=31,963). We excluded patients who died or were discharged within 24 hr after admission (N=2,466), yielding a total study population of 30,386 patients. Patients referred to the Georges Pompidou European Hospital (HEGP) were not included in our analysis because of unreliable data collection. The data analysis was based on a database extract dated July 27, 2023.

Hospitalizations related to confirmed COVID-19 were identified as in-hospital stays lasting > 24 hr and starting within a time window of -7/+15 days (+/-ICD10 code U07.1) with a positive COVID-19 result from RT-PCR analysis. The date of inclusion in the study cohort (index date) was defined as the date of hospital admission. The end of follow-up was defined by the time of death, discharge home, day-90 after admission, whichever occurred first, or administrative censoring on June 1, 2022.

**Ethical considerations**

The study was performed in accordance with the Declaration of Helsinki and was approved by the institutional review board (authorization no IRB 00011591) of the AP-HP CDW (ref CSE 20-11_COVIPREDS). The CDW was itself authorized by a decision of the French Regulatory Agency (CNIL no. 1980120). Individuals who objected to the use of their data were excluded from this study in accordance with the French legislation.

**Data collection**

Identification of comorbidities in electronic health records (EHRs) involved specific codes from the ICD-10, collected up to 5 years before the COVID-19–related stay (**Supplemental Table 1**) and using reference coding instructions for Elixhauser and Charlson comorbidities indices (Quan, Sundararajan et al. 2005). The following demographic characteristics, comorbidities and risk factors were collected: age, sex, body mass index (as a continuous variable), obesity (identified from EHRs), diabetes, hypertension, congestive heart failure, myocardial infarction, cardiac arrythmia, valvular disease, peripheral vascular disease, coagulopathy, rheumatoid arthritis/collagen vas, organ transplant, chronic pulmonary disease, pulmonary circulation disease, renal failure, liver disease, cerebrovascular disease, paralysis, dementia, other neurological disorders, solid tumor without metastasis, metastatic cancer, lymphoma, acquired immune deficiency syndrome.

The following blood biological features were also recorded, selecting the earliest measurement in the 3 days following hospital admission: arterial blood gas analysis (with oxygen saturation and partial pressure, carbon dioxide partial pressure, pH, bicarbonates, and lactate), glycemia, blood serum ionogram (with sodium, potassium, and calcium), hemoglobin, complete blood count (with leukocytes, lymphocytes, neutrophils, eosinophils, and platelets), troponin, lactate dehydrogenase, creatine kinase, creatinine, urea, partial thromboplastin time, prothrombin time, D-Dimers, C-reactive protein, fibrinogen, procalcitonin, alanine transaminase, and serum bicarbonates.

As the main exposure of interest, parental status was extracted from EHR text reports using regular expressions accounting for typos and spelling errors, tailored to identify French words relating to having one or multiple children (“with children”) or explicitly none (“explicitly without children”); if no mention of children or absence of children was found, patients were classified as “without children”. To study the validity of this automatically extracted parental status variable, 100 EHRs (50 identified “with children”, 25 “explicitly without children”, 25 “without children”) were randomly sampled from the patients included in the analysis, and a manual verification of all available text reports was performed independently by EA and JR. Among the 100 EHRs extracted, 3 errors were identified (97% predictive positive value [PPV]), with 1 EHR without children mention among the 50 “with children” EHRs (98% PPV), 1 EHR with a child mentioned among the “explicitly without children” EHRs (96% PPV), and 1 EHR with a child mentioned among the 25 “without children” EHRs (96% PPV).

Patient outcomes including intensive care unit (ICU) admission, in-hospital death and death at 90 days after admission were collected from EHR data from the AP-HP CDW, in which information on patient mortality is regularly updated from the French National Institute for Statistics and Economic Studies (INSEE).

**Statistical analysis**

### *Descriptive analyses*

Descriptive results are given as median (interquartile range [IQR]) for continuous variables, and number (%) for categorical variables. Unadjusted between-group comparisons involved the chi-squared test for categorical variables and t tests and Mann–Whitney rank sum tests for continuous variables, as appropriate. For time-to-event analyses, Kaplan-Meier survival curves were plotted at the 90-day time horizon.

### *Survival analyses*

As the primary endpoint of the study, 90-day mortality was analyzed as a time-to-event endpoint under a survival analysis approach, computing hazard ratios (HRs) by Cox proportional-hazards modeling, along with their corresponding 95% confidence intervals (95% CIs). To account for the confounding effects of baseline characteristics in the relation between parental status and all-cause mortality, all analyses were adjusted for a broad selection of prognostic factors, including a comprehensive set of patient demographics, comorbidities and time period of admission, as detailed in the data collection section. Analyses were conducted on the whole study period, and after stratifying by COVID-19 waves, defined as follows: wave#1 from January 29 to July 6, 2020, wave#2 from July 7, 2020 to January 4, 2021, wave#3 from January 5 to July 5, 2021, wave#4: from July 6 to November 10, 2021, wave#5 from November 11, 2021 to March 1, 2022. Because contact with children likely depends on the age of patients and to avoid the limitations of dichotomization, an interaction between age and parental status was evaluated using age as a continuous variable. We compared model fits using linear terms and restricted cubic splines (3–6 knots). Model selection was primarily guided by the Bayesian Information Criterion (BIC) to favour parsimony, as well as the Akaike Information Criterion (AIC) and the Likelihood Ratio Test (LRT). Analyses were further stratified by age (median <65 vs. ≥65 years old). Unadjusted Kaplan-Meier curves were plotted as were adjusted survival curves using the Stata stpm2 command based on flexible parametric modeling (Lambert and Royston 2009).

### *Missing data*

Most variables had from 7% to 30% missing data, except for glycemia, troponin, procalcitonin, LDH and BGA:PaO2, with missing information rates >50%. To reduce potential selection bias arising from complete-case analyses, all analyses were performed after missing data imputation using *missForest*, a nonparametric method based on random forest imputation that accommodates non-linearities and interactions (Stekhoven and Bühlmann 2012). Imputation quality was assessed using out-of-bag (OOB) error estimates provided by the missForest algorithm. The final imputation yielded a Normalized Root Mean Squared Error (NRMSE) of 0.377 for continuous variables (none of the categorical variables - including gender and comorbidities - were imputed). Data were assumed to be missing at random, conditional on the outcome and other predictors.. Descriptive statistics before-after missing data imputation are given in **Supplemental Table 2**. A sensitivity analysis after discarding variables with missing data rates >30% was also performed to check the robustness of the findings.

*Normality assessment*

Continuous variables were assessed for normality through visual inspection of histograms and Q-Q plots. Variables with highly skewed distributions were log-transformed prior to imputation to improve algorithm performance; however, raw values were retained in the primary models to facilitate clinical interpretation, with log-transformed sensitivity analyses conducted to ensure consistency.

All analyses were performed at the two-tailed P<0.05 level, using Python 3.7 for data collection and preprocessing, and R 4.4.1 for data imputation and multivariate analyses (R Foundation for Statistical Computing, Vienna, Austria; packages *missForest, survival*).

**References**

- Chouchana L, Beeker N, Garcelon N, et al. Association of antihypertensive agents with the risk of in-hospital death in patients with COVID-19. *Cardiovasc Drugs Ther.* 2021;1-6.
- Lambert PC, Royston P. Further development of flexible parametric models for survival analysis. *Stata J.* 2009;9(2):265-290.
- Lombardi Y, Azoyan L, Szychowiak P, et al. External validation of prognostic scores for COVID-19: a multicenter cohort study of patients hospitalized in Greater Paris University Hospitals. *Intensive Care Med.* 2021;47(12):1426-1439.
- Quan H, Sundararajan V, Halfon P, et al. Coding algorithms for defining comorbidities in ICD-9-CM and ICD-10 administrative data. *Med Care.* 2005;43(11):1130-1139.
- Stekhoven DJ, Bühlmann P. MissForest—non-parametric missing value imputation for mixed-type data. *Bioinformatics.* 2012;28(1):112-118.

**Supplemental Table 1. List of ICD-10 codes used to extract patient comorbidities**

| **Comorbidity name** | **List of associated ICD-10 codes** |
| --- | --- |
| **Diabetes* ^e^** | E10.0, E10.1, E10.9, E11.0, E11.1, E11.9, E12.0, E12.1, E12.9, E13.0, E13.1, E13.9, E14.0, E14.1, E14.9, E10.2 - E10.8, E11.2 - E11.8, E12.2 - E12.8, E13.2 - E13.8, E14.2 - E14.8 |
| **Obesity* ^e^** | E66.x |
| **Hypertension* ^e^** | I10.x, I11.x - I13.x, I15.x |
| **Congestive heart failure*^e^** | I09.9, I11.0, I13.0, I13.2, I25.5, I42.0, I42.5 - I42.9, I43.x, I50.x, P29.0 |
| **Myocardial infarction* ^C^** | I21.x, I22.x, I25.2 |
| **Cardiac arrhythmias* ^e^** | I44.1 - I44.3, I45.6, I45.9, I47.x - I49.x, R00.0, R00.1, R00.8, T82.1, Z45.0, Z95.0 |
| **Valvular disease* ^e^** | A52.0, I05.x - I08.x, I09.1, I09.8, I34.x - I39.x, Q23.0 - Q23.3, Z95.2 - Z95.4 |
| **Peripheral vascular disorders* ^e^** | I70.x, I71.x, I73.1, I73.8, I73.9, I77.1, I79.0, I79.2, K55.1, K55.8, K55.9, Z95.8, Z95.9 |
| **Coagulopathy* ^e^** | D65 - D68.x, D69.1, D69.3 - D69.6 |
| **Rheumatoid arthritis/collagen vascular diseases* ^e^** | L94.0, L94.1, L94.3, M05.x, M06.x, M08.x, M12.0, M12.3, M30.x, M31.0 - M31.3, M32.x - M35.x, M45.x, M46.1, M46.8, M46. |
| **Transplants** | Z94.0, Z94.1, Z94.2, Z94.3, Z94.4, Z94.8 |
| **Chronic pulmonary disease* ^e^** | I27.8, I27.9, J40.x - J47.x, J60.x - J67.x, J68.4, J70.1, J70.3 |
| **Pulmonary circulation disorders* ^e^** | I26.x, I27.x, I28.0, I28.8, I28.9 |
| **Renal failure* ^e^** | I12.0, I13.1, N18.x, N19.x, N25.0, Z49.0 - Z49.2, Z94.0, Z99.2 |
| **Liver disease* ^e^** | B18.x, I85.x, I86.4, I98.2, K70.x, K71.1, K71.3 - K71.5, K71.7, K72.x - K74.x, K76.0, K76.2 - K76.9, Z94.4 |
| **Cerebrovascular disease* ^C^** | G45.x, G46.x, H34.0, I60.x - I69.x |
| **Paralysis* ^e^** | G04.1, G11.4, G80.1, G80.2, G81.x, G82.x, G83.0 - G83.4, G83.9 |
| **Dementia* ^C^** | F10, E52, G62.1, I42.6, K29.2, K70.0, K70.3, K70.9, T51.x, Z50.2, Z71.4, Z72.1 |
| **Other neurological disorders* ^e^** | G10.x - G13.x, G20.x - G22.x, G25.4, G25.5, G31.2, G31.8, G31.9, G32.x, G35.x - G37.x, G40.x, G41.x, G93.1, G93.4, R47.0, R56.x |
| **Solid tumour without metastasis* ^e^** | C00.x - C26.x, C30.x - C34.x, C37.x - C41.x, C43.x, C45.x - C58.x, C60.x - C76.x, C97.x |
| **Metastatic cancer* ^e^** | C77.x - C80.x |
| **Lymphoma* ^e^** | C81.x - C85.x, C88.x, C96.x, C90.0, C90.2 |
| **AIDS/HIV* ^e^** | B20.x - B22.x, B24.x |
| *.x means all the sub-codes from the main ICD-10 code* | |
| *Source: *Quan et al. “Coding Algorithms for Defining Comorbidities in ICD-9-CM and ICD-10 Administrative Data”. ^E^: Elixhauser index, ^C^: Charlson Index* | |

**Supplemental Table 2**. Descriptive statistics of variables with missing information, before-after missing data imputation

|  | Raw data | | Imputed data | |  |
| --- | --- | --- | --- | --- | --- |
| **Characteristic** | N  complete (%) | N = 30386^1^ | N  complete (%) | N = 30386^1^ |  |
| Body mass index, kg/m² | 9,350 (31%) | 28.0 ±6.5 | 30,386 (100%) | 27.2 ±4.23 |  |
| Na, mmol/L | 28,096 (92%) | 136 [134;139] | 30,386 (100%) | 137 [134;139] |  |
| K, mmol/L | 27,669 (91%) | 4.09 [3.70;4.42] | 30,386 (100%) | 4.06 [3.78;4.40] |  |
| Hemoglobin, g/dL | 26,644 (88%) | 12.9 [11.5;14.2] | 30,386 (100%) | 12.8 [11.6;14.1] |  |
| Leukocytes count, x 10^9 / L | 26,643 (88%) | 6.8 [5.0;9.4] | 30,386 (100%) | 7.0 [5.2;9.2] |  |
| Lymphocyte count, x 10^9 / L | 25,904 (85%) | 0.96 [0.66;1.38] | 30,386 (100%) | 1.03 [0.70;1.41] |  |
| Neutrophil count, x 10^9 / L | 26,004 (86%) | 5.0 [3.44;7.5] | 30,386 (100%) | 5.2 [3.65;7.3] |  |
| Eosinophil count, x 10^9 / L | 25,899 (85%) | 0.00 [0.00;0.03] | 30,386 (100%) | 0.01 [0.00;0.05] |  |
| Platelet count, x 10^9 / L | 26,625 (88%) | 212 [163;275] | 30,386 (100%) | 219 [170;269] |  |
| Serum creatinine, µmol/L | 28,120 (93%) | 81.0 [64.0;110] | 30,386 (100%) | 79.0 [62.0;107] |  |
| Urea, mmol/L | 27,520 (91%) | 6.3 [4.42;9.8] | 30,386 (100%) | 6.1 [4.30;9.3] |  |
| C-reactive protein (mg/L) | 26,077 (86%) | 67.6 [25.0;130] | 30,386 (100%) | 59.8 [20.0;121] |  |
| Protides, g/L | 27,291 (90%) | 71.0 [66.0;76.0] | 30,386 (100%) | 71.0 [66.8;75.0] |  |
| Alanine transaminase, U/L | 24,708 (81%) | 29.0 [19.0;48.0] | 30,386 (100%) | 27.0 [19.4;43.0] |  |
| Bilirubin total, µmol/L | 24,899 (82%) | 8.4 [6.0;12.0] | 30,386 (100%) | 8.6 [6.5;11.8] |  |
| Partial thromboplastin time | 24,155 (79%) | 1.12 [1.01;1.26] | 30,386 (100%) | 1.12 [1.03;1.24] |  |
| Prothrombin time (%) | 24,988 (82%) | 87.0 [75.0;98.0] | 30,386 (100%) | 87.0 [76.8;96.0] |  |
| Glycemia, mmol/L | 9,668 (32%) | 6.6 [5.6;9.0] | 30,386 (100%) | 7.1 [6.1;8.6] |  |
| HCO3- serum, mmol/L | 17,238 (57%) | 24.0 [21.2;26.2] | 30,386 (100%) | 24.0 [22.0;26.0] |  |
| Calcium, mmol/L | 18,334 (60%) | 2.20 [2.09;2.30] | 30,386 (100%) | 2.22 [2.15;2.29] |  |
| Fibrinogen, g/L | 20,446 (67%) | 5.6 [4.54;6.8] | 30,386 (100%) | 5.4 [4.50;6.4] |  |
| Procalcitonin, µg/L | 14,509 (48%) | 0.18 [0.09;0.49] | 30,386 (100%) | 0.15 [0.09;0.34] |  |
| Troponin, ng/L | 13,642 (45%) | 15.0 [9.6;35.4] | 30,386 (100%) | 15.0 [9.3;30.8] |  |
| Lactate dehydrogenase, U/L | 14,558 (48%) | 379 [277;529] | 30,386 (100%) | 363 [280;474] |  |
| Creatine kinase, U/L | 15,607 (51%) | 134 [67.0;319] | 30,386 (100%) | 119 [77.0;217] |  |
| D-Dimer (µg/L) | 17,111 (56%) | 1,060 [626;1,934] | 30,386 (100%) | 1,107 [744;1,674] |  |
| Arterial blood gas test: SpO2, % | 15,160 (50%) | 94.9 [92.0;97.1] | 30,386 (100%) | 94.4 [92.3;95.8] |  |
| Arterial blood gas test: PaO2, mmHg | 14,455 (48%) | 71.0 [57.0;87.6] | 30,386 (100%) | 73.3 [63.1;81.3] |  |
| Arterial blood gas test: PaCO2, mmHg | 15,304 (50%) | 35.0 [30.5;39.3] | 30,386 (100%) | 35.2 [32.1;38.1] |  |
| Arterial blood gas test: HCO3-, mmol/L | 14,935 (49%) | 24.7 [22.3;26.9] | 30,386 (100%) | 24.7 [23.1;26.2] |  |
| Arterial blood gas test: lactate, mmol/L | 15,703 (52%) | 1.20 [0.90;1.70] | 30,386 (100%) | 1.17 [0.96;1.50] |  |
| Arterial blood gas test: pH | 15,289 (50%) | 7.4 [7.4;7.5] | 30,386 (100%) | 7.4 [7.4;7.5] |  |
| ^1^Mean ±SD; median [25%;75%] | | | | | |

**Supplemental Table 3**. Detailed characteristics of patients with COVID-19 and without and with children over the whole study period

| **Characteristic** | **Overall**  N = 30386^1^ | **Without children**  N=7673^1^ | **With children**  N=22713^1^ | **p-value**^2^ | |
| --- | --- | --- | --- | --- | --- |
| ***Demographics*** |  |  |  |  | |
| Age, years | 63.7 ±19.1 | 56.3 ±21.5 | 66.2 ±17.5 | **<0.001** | |
| Male sex | 16,801 (55.3%) | 4,442 (57.9%) | 12,359 (54.4%) | **<0.001** | |
| ***Outcomes*** |  |  |  |  | |
| 28-day mortality | 4,572 (15.0%) | 892 (11.6%) | 3,680 (16.2%) | **<0.001** | |
| 90-day mortality | 5,711 (18.8%) | 1,152 (15.0%) | 4,559 (20.1%) | **<0.001** | |
| In hospital mortality | 4,917 (16.2%) | 995 (13.0%) | 3,922 (17.3%) | **<0.001** | |
| ICU transfer | 8,960 (29.5%) | 2,115 (27.6%) | 6,845 (30.1%) | **<0.001** | |
| Length of stay, days | 9.0 [5.0;16.0] | 8.0 [4.00;15.0] | 9.0 [5.0;17.0] | **<0.001** | |
| ***Comorbidities*** |  |  |  |  | |
| Number of comorbidities | 2.00 [1.00;4.00] | 1.00 [0.00;3.00] | 2.00 [1.00;4.00] | **<0.001** | |
| Diabetes | 8,199 (27.0%) | 1,346 (17.5%) | 6,853 (30.2%) | **<0.001** | |
| Obesity | 4,876 (16.0%) | 1,013 (13.2%) | 3,863 (17.0%) | **<0.001** | |
| Body mass index, kg/m² | 27.2 ±4.23 | 27.0 ±4.28 | 27.3 ±4.21 | **<0.001** | |
| Hypertension | 12,986 (42.7%) | 2,316 (30.2%) | 10,670 (47.0%) | **<0.001** | |
| Congestive heart failure | 5,073 (16.7%) | 885 (11.5%) | 4,188 (18.4%) | **<0.001** | |
| Myocardial infarction | 1,113 (3.7%) | 189 (2.5%) | 924 (4.1%) | **<0.001** | |
| Cardiac arrythmia | 6,024 (19.8%) | 1,131 (14.7%) | 4,893 (21.5%) | **<0.001** | |
| Valvular disease | 1,398 (4.6%) | 222 (2.9%) | 1,176 (5.2%) | **<0.001** | |
| Peripheral vascular disease | 2,100 (6.9%) | 350 (4.6%) | 1,750 (7.7%) | **<0.001** | |
| Coagulopathy | 2,252 (7.4%) | 438 (5.7%) | 1,814 (8.0%) | **<0.001** | |
| Rheumatoid arthritis/collagen vas | 1,078 (3.5%) | 211 (2.7%) | 867 (3.8%) | **<0.001** | |
| Organ transplant | 1,285 (4.2%) | 221 (2.9%) | 1,064 (4.7%) | **<0.001** | |
| Chronic pulmonary disease | 3,927 (12.9%) | 833 (10.9%) | 3,094 (13.6%) | **<0.001** | |
| Pulmonary circulation disease | 2,715 (8.9%) | 561 (7.3%) | 2,154 (9.5%) | **<0.001** | |
| Renal failure | 4,815 (15.8%) | 845 (11.0%) | 3,970 (17.5%) | **<0.001** | |
| Liver disease | 2,196 (7.2%) | 456 (5.9%) | 1,740 (7.7%) | **<0.001** | |
| Cerebrovascular disease | 3,183 (10.5%) | 561 (7.3%) | 2,622 (11.5%) | **<0.001** | |
| Paralysis | 1,670 (5.5%) | 346 (4.5%) | 1,324 (5.8%) | **<0.001** | |
| Dementia | 3,944 (13.0%) | 775 (10.1%) | 3,169 (14.0%) | **<0.001** | |
| Other neurological disorders | 3,080 (10.1%) | 702 (9.1%) | 2,378 (10.5%) | **<0.001** | |
| Solid tumor without metastasis | 3,085 (10.2%) | 612 (8.0%) | 2,473 (10.9%) | **<0.001** | |
| Metastatic cancer | 1,256 (4.1%) | 235 (3.1%) | 1,021 (4.5%) | **<0.001** | |
| Lymphoma | 845 (2.8%) | 140 (1.8%) | 705 (3.1%) | **<0.001** | |
| Acquired immune deficiency syndrome | 490 (1.6%) | 144 (1.9%) | 346 (1.5%) | **0.034** | |
| ***Biological parameters*** |  |  |  |  | |
| Na, mmol/L | 137 [134;139] | 137 [134;139] | 136 [134;139] | **<0.001** | |
| K, mmol/L | 4.06 [3.78;4.40] | 4.00 [3.77;4.30] | 4.09 [3.79;4.40] | **<0.001** | |
| Hemoglobin, g/dL | 12.8 [11.6;14.1] | 13.1 [11.8;14.3] | 12.8 [11.6;14.0] | **<0.001** | |
| Leukocyte count, x 10^9 / L | 7.0 [5.2;9.2] | 7.3 [5.4;9.7] | 7.0 [5.2;9.1] | **<0.001** | |
| Lymphocyte count, x 10^9 / L | 1.03 [0.70;1.41] | 1.07 [0.73;1.51] | 1.01 [0.70;1.38] | **<0.001** | |
| Neutrophil count, x 10^9 / L | 5.2 [3.65;7.3] | 5.4 [3.78;7.6] | 5.1 [3.61;7.2] | **<0.001** | |
| Eosinophil count, x 10^9 / L | 0.01 [0.00;0.05] | 0.01 [0.00;0.06] | 0.01 [0.00;0.04] | **<0.001** | |
| Platelet count, x 10^9 / L | 219 [170;269] | 224 [176;274] | 217 [168;267] | **<0.001** | |
| Serum creatinine, µmol/L | 79.0 [62.0;107] | 75.7 [60.0;98.0] | 81.0 [63.0;109] | **<0.001** | |
| Urea, mmol/L | 6.1 [4.30;9.3] | 5.5 [3.80;8.4] | 6.3 [4.50;9.6] | **<0.001** | |
| C reactive protein (mg/L) | 59.8 [20.0;121] | 49.6 [15.0;109] | 63.0 [22.6;125] | **<0.001** | |
| Protides, g/L | 71.0 [66.8;75.0] | 71.3 [67.0;75.6] | 71.0 [66.3;75.0] | **<0.001** | |
| Alanine transaminase, U/L | 27.0 [19.4;43.0] | 28.0 [20.0;44.6] | 27.0 [19.1;42.5] | **<0.001** | |
| Bilirubin total, µmol/L | 8.6 [6.5;11.8] | 8.8 [6.8;12.0] | 8.5 [6.4;11.7] | **<0.001** | |
| Partial thromboplastin time | 1.12 [1.03;1.24] | 1.11 [1.02;1.22] | 1.12 [1.03;1.24] | **<0.001** | |
| Prothrombin time (%) | 87.0 [76.8;96.0] | 88.0 [78.0;96.6] | 87.0 [76.0;96.0] | **<0.001** | |
| Glycemia, mmol/L | 7.1 [6.1;8.6] | 6.8 [5.9;8.1] | 7.2 [6.2;8.8] | **<0.001** | |
| HCO3- serum, mmol/L | 24.0 [22.0;26.0] | 24.0 [22.0;25.9] | 24.0 [22.0;26.0] | 0.53 | |
| Calcium, mmol/L | 2.22 [2.15;2.29] | 2.23 [2.16;2.30] | 2.22 [2.14;2.28] | **<0.001** | |
| Fibrinogen, g/L | 5.4 [4.50;6.4] | 5.2 [4.36;6.3] | 5.4 [4.57;6.5] | **<0.001** | |
| Procalcitonin, µg/L | 0.15 [0.09;0.34] | 0.14 [0.08;0.30] | 0.16 [0.09;0.35] | **<0.001** | |
| Troponin, ng/L | 15.0 [9.3;30.8] | 13.0 [7.9;25.2] | 16.0 [10.0;32.3] | **<0.001** | |
| Lactate dehydrogenase, U/L | 363 [280;474] | 352 [266;472] | 366 [283;474] | **<0.001** | |
| Creatine kinase, U/L | 119 [77.0;217] | 120 [79.0;225] | 119 [76.4;215] | **0.001** | |
| D-Dimers (µg/L) | 1,107 [744;1,674] | 1,090 [712;1,673] | 1,113 [756;1,674] | **0.008** | |
| Arterial blood gas test: SpO2, % | 94.4 [92.3;95.8] | 94.5 [92.6;95.9] | 94.4 [92.2;95.8] | **<0.001** | |
| Arterial blood gas test: PaO2, mmHg | 73.3 [63.1;81.3] | 74.1 [64.6;82.3] | 73.1 [62.7;81.0] | **<0.001** | |
| Arterial blood gas test: PaCO2, mmHg | 35.2 [32.1;38.1] | 35.2 [32.2;38.1] | 35.2 [32.0;38.1] | 0.54 | |
| Arterial blood gas test: HCO3-, mmol/L | 24.7 [23.1;26.2] | 24.6 [23.1;26.0] | 24.7 [23.1;26.2] | **0.012** | |
| Arterial blood gas test: lactate, mmol/L | 1.17 [0.96;1.50] | 1.17 [0.98;1.50] | 1.17 [0.96;1.50] | 0.32 | |
| Arterial blood gas test: pH | 7.4 [7.4;7.5] | 7.4 [7.4;7.5] | 7.4 [7.4;7.5] | **<0.001** | |
| ^1^Mean ±SD; n (%); median [25%;75%] | | | | |  |
| ^2^Welch two sample t-test; Pearson's chi-squared test; Wilcoxon rank sum test  ICU, intensive care unit | | | | |  |

**Supplemental Table 4.** Results from univariate and multivariate Cox proportional-hazards modeling for 90-day overall survival: whole study population

|  | Univariable analysis | | Multivariable analysis | |
| --- | --- | --- | --- | --- |
| **Characteristic** | **HR (95% CI)**^1^ | **p-value** | **HR (95% CI)**^2^ | **p-value** |
| Parental status |  |  |  |  |
| Without children | — |  | — |  |
| With children | 1.38 (1.29 ; 1.47) | **<0.001** | 1.004 (0.94 ; 1.07) | 0.91 |
| Period (COVID-19 wave) |  | **<0.001** |  |  |
| 1 | — |  | — |  |
| 2 | 0.77 (0.71 ; 0.83) | **<0.001** | 0.78 (0.72 ; 0.84) | **<0.001** |
| 3 | 0.77 (0.72 ; 0.83) | **<0.001** | 0.84 (0.79 ; 0.90) | **<0.001** |
| 4 | 0.63 (0.56 ; 0.72) | **<0.001** | 0.82 (0.72 ; 0.93) | **0.002** |
| 5 | 0.66 (0.61 ; 0.71) | **<0.001** | 0.75 (0.69 ; 0.81) | **<0.001** |
| Age, years | 1.05 (1.04 ; 1.05) | **<0.001** | 1.06 (1.06 ; 1.06) | **<0.001** |
| Male sex | 1.38 (1.31 ; 1.46) | **<0.001** | 1.27 (1.20 ; 1.35) | **<0.001** |
| Number of comorbidities | 1.13 (1.12 ; 1.14) | **<0.001** | 0.90 (0.70 ; 1.15) | 0.41 |
| Diabetes | 1.21 (1.14 ; 1.28) | **<0.001** | 0.95 (0.73 ; 1.23) | 0.67 |
| Obesity | 0.80 (0.75 ; 0.87) | **<0.001** | 1.06 (0.82 ; 1.39) | 0.65 |
| Body mass index, kg/m² | 0.97 (0.96 ; 0.98) | **<0.001** | 1.02 (1.01 ; 1.03) | **<0.001** |
| Hypertension | 1.51 (1.43 ; 1.59) | **<0.001** | 0.94 (0.73 ; 1.22) | 0.65 |
| Congestive heart failure | 1.89 (1.78 ; 2.00) | **<0.001** | 1.14 (0.88 ; 1.48) | 0.34 |
| Myocardial infarction | 1.45 (1.29 ; 1.63) | **<0.001** | 1.10 (0.83 ; 1.46) | 0.49 |
| Cardiac arrythmia | 1.83 (1.73 ; 1.94) | **<0.001** | 1.10 (0.85 ; 1.43) | 0.45 |
| Valvular disease | 1.54 (1.39 ; 1.71) | **<0.001** | 1.04 (0.79 ; 1.37) | 0.77 |
| Peripheral vascular disease | 1.50 (1.37 ; 1.63) | **<0.001** | 1.05 (0.80 ; 1.36) | 0.74 |
| Coagulopathy | 1.51 (1.39 ; 1.64) | **<0.001** | 1.09 (0.84 ; 1.43) | 0.51 |
| Rheumatoid arthritis/collagen vas | 1.010 (0.88 ; 1.16) | 0.89 | 1.34 (1.006 ; 1.78) | **0.045** |
| Organ transplant | 1.24 (1.10 ; 1.39) | **<0.001** | 1.73 (1.31 ; 2.30) | **<0.001** |
| Chronic pulmonary disease | 1.18 (1.10 ; 1.27) | **<0.001** | 1.16 (0.89 ; 1.51) | 0.27 |
| Pulmonary circulation disease | 1.38 (1.27 ; 1.49) | **<0.001** | 1.16 (0.89 ; 1.50) | 0.28 |
| Renal failure | 1.80 (1.69 ; 1.91) | **<0.001** | 1.13 (0.87 ; 1.46) | 0.37 |
| Liver disease | 1.13 (1.03 ; 1.24) | **0.013** | 1.23 (0.94 ; 1.62) | 0.14 |
| Cerebrovascular disease | 1.38 (1.28 ; 1.49) | **<0.001** | 1.06 (0.81 ; 1.38) | 0.67 |
| Paralysis | 1.02 (0.91 ; 1.14) | 0.73 | 1.04 (0.78 ; 1.37) | 0.80 |
| Dementia | 1.69 (1.58 ; 1.81) | **<0.001** | 0.96 (0.74 ; 1.25) | 0.79 |
| Other neurological disorders | 1.38 (1.28 ; 1.50) | **<0.001** | 1.36 (1.04 ; 1.76) | **0.024** |
| Solid tumor without metastasis | 1.62 (1.50 ; 1.74) | **<0.001** | 1.09 (0.83 ; 1.42) | 0.54 |
| Metastatic cancer | 2.05 (1.86 ; 2.26) | **<0.001** | 2.11 (1.60 ; 2.79) | **<0.001** |
| Lymphoma | 1.72 (1.51 ; 1.95) | **<0.001** | 1.46 (1.10 ; 1.94) | **0.009** |
| Acquired immune deficiency syndrome | 0.66 (0.52 ; 0.85) | **0.001** |  |  |
| Na, mmol/L | 1.010 (1.004 ; 1.02) | **0.002** | 1.01 (1.006 ; 1.02) | **<0.001** |
| K, mmol/L | 1.39 (1.34 ; 1.45) | **<0.001** | 1.08 (1.04 ; 1.13) | **<0.001** |
| Hemoglobin, g/dL | 0.92 (0.91 ; 0.94) | **<0.001** | 0.97 (0.96 ; 0.99) | **<0.001** |
| Leukocyte count, x 10^9 / L | 1.03 (1.02 ; 1.03) | **<0.001** | 0.99 (0.97 ; 1.004) | 0.14 |
| Lymphocyte count, x 10^9 / L | 0.85 (0.82 ; 0.89) | **<0.001** | 1.007 (0.99 ; 1.03) | 0.52 |
| Neutrophil count, x 10^9 / L | 1.05 (1.04 ; 1.05) | **<0.001** | 1.02 (1.007 ; 1.04) | **0.005** |
| Eosinophil count, x 10^9 / L | 0.02 (0.01 ; 0.03) | **<0.001** | 0.19 (0.12 ; 0.31) | **<0.001** |
| Platelet count, x 10^9 / L | 0.997 (0.997 ; 0.998) | **<0.001** | 0.998 (0.998 ; 0.999) | **<0.001** |
| Serum creatinine, µmol/L | 1.002 (1.001 ; 1.002) | **<0.001** | 0.999 (0.999 ; 1.000) | **<0.001** |
| Urea, mmol/L | 1.05 (1.05 ; 1.05) | **<0.001** | 1.02 (1.02 ; 1.03) | **<0.001** |
| C-reactive protein (mg/L) | 1.004 (1.004 ; 1.004) | **<0.001** | 1.003 (1.002 ; 1.003) | **<0.001** |
| Protides, g/L | 0.96 (0.96 ; 0.97) | **<0.001** | 0.99 (0.99 ; 0.994) | **<0.001** |
| Alanine transaminase, U/L | 1.000 (1.000 ; 1.001) | 0.43 | 0.997 (0.997 ; 0.998) | **<0.001** |
| Bilirubin total, µmol/L | 1.006 (1.005 ; 1.006) | **<0.001** | 1.004 (1.003 ; 1.006) | **<0.001** |
| Partial thromboplastin time (PTT) | 1.63 (1.56 ; 1.70) | **<0.001** | 1.16 (1.09 ; 1.23) | **<0.001** |
| Prothrombin time (%) | 0.98 (0.98 ; 0.98) | **<0.001** | 0.997 (0.996 ; 0.999) | **0.002** |
| Glycemia, mmol/L | 1.09 (1.08 ; 1.10) | **<0.001** | 1.04 (1.03 ; 1.05) | **<0.001** |
| HCO3- serum, mmol/L | 0.92 (0.91 ; 0.93) | **<0.001** | 0.96 (0.95 ; 0.97) | **<0.001** |
| Calcium, mmol/L | 0.16 (0.14 ; 0.18) | **<0.001** | 0.50 (0.42 ; 0.61) | **<0.001** |
| Fibrinogen, g/L | 1.09 (1.07 ; 1.10) | **<0.001** | 0.94 (0.92 ; 0.96) | **<0.001** |
| Procalcitonin, µg/L | 1.06 (1.06 ; 1.07) | **<0.001** | 0.994 (0.99 ; 1.002) | 0.12 |
| Troponin, ng/L | 1.000 (1.000 ; 1.000) | **<0.001** | 1.000 (1.000 ; 1.000) | **<0.001** |
| Lactate dehydrogenase, U/L | 1.002 (1.002 ; 1.002) | **<0.001** | 1.002 (1.002 ; 1.002) | **<0.001** |
| Creatine kinase, U/L | 1.000 (1.000 ; 1.000) | **<0.001** | 1.000 (1.000 ; 1.000) | **0.013** |
| D-Dimer (µg/L) | 1.000 (1.000 ; 1.000) | **<0.001** | 1.000 (1.000 ; 1.000) | 0.076 |
| Arterial blood gas test: SPO2, % | 0.97 (0.97 ; 0.98) | **<0.001** | 0.99 (0.99 ; 0.992) | **<0.001** |
| Arterial blood gas test: PaO2, mmHg | 1.000 (0.999 ; 1.001) | 0.45 | 0.999 (0.998 ; 1.000) | 0.12 |
| Arterial blood gas test: PaCO2, mmHg | 0.995 (0.993 ; 0.998) | **0.002** | 0.994 (0.990 ; 0.998) | **0.001** |
| Arterial blood gas test: HCO3-, mmol/L | 0.92 (0.92 ; 0.93) | **<0.001** | 1.04 (1.03 ; 1.06) | **<0.001** |
| Arterial blood gas test: lactate, mmol/L | 1.24 (1.22 ; 1.25) | **<0.001** | 1.05 (1.02 ; 1.07) | **<0.001** |
| Arterial blood gas test: pH | 0.02 (0.01 ; 0.03) | **<0.001** | 0.06 (0.03 ; 0.10) | **<0.001** |
| ^1^Hazard ratio (95% confidence interval) from unadjusted Cox proportional-hazards regression modeling. | | | | |
| ^2^Hazard ratio (95% confidence interval) from multivariable Cox proportional-hazards regression modeling. | | | | |

**Supplemental Table 5**. Results from univariate and multivariate Cox proportional-hazards modeling for 90-day overall survival: patients < 65 years old

|  | Univariable analysis | | Multivariable analysis | |
| --- | --- | --- | --- | --- |
| **Characteristic** | **HR (95% CI)**^1^ | **p-value** | **HR (95% CI)**^2^ | **p-value** |
| Parental status |  |  |  |  |
| Without children | — |  | — |  |
| With children | 1.27 (1.12 ; 1.45) | **<0.001** | 0.83 (0.72 ; 0.95) | **0.009** |
| Period (COVID-19 wave) |  | **<0.001** |  |  |
| 1 | — |  | — |  |
| 2 | 0.68 (0.56 ; 0.82) | **<0.001** | 0.74 (0.61 ; 0.89) | **0.002** |
| 3 | 0.69 (0.59 ; 0.80) | **<0.001** | 0.75 (0.64 ; 0.88) | **<0.001** |
| 4 | 0.63 (0.49 ; 0.81) | **<0.001** | 0.85 (0.66 ; 1.09) | 0.21 |
| 5 | 0.63 (0.53 ; 0.75) | **<0.001** | 0.86 (0.72 ; 1.04) | 0.12 |
| Age, years | 1.07 (1.06 ; 1.08) | **<0.001** | 1.06 (1.05 ; 1.06) | **<0.001** |
| Male sex | 1.97 (1.73 ; 2.25) | **<0.001** | 1.34 (1.15 ; 1.55) | **<0.001** |
| Number of comorbidities | 1.26 (1.24 ; 1.29) | **<0.001** | 0.99 (0.71 ; 1.38) | 0.95 |
| Diabetes | 1.72 (1.52 ; 1.96) | **<0.001** | 0.81 (0.55 ; 1.17) | 0.26 |
| Obesity | 1.35 (1.18 ; 1.55) | **<0.001** | 0.95 (0.66 ; 1.38) | 0.81 |
| Body mass index, kg/m² | 1.02 (1.01 ; 1.03) | **<0.001** | 1.03 (1.01 ; 1.04) | **<0.001** |
| Hypertension | 1.90 (1.68 ; 2.14) | **<0.001** | 0.82 (0.57 ; 1.19) | 0.30 |
| Congestive heart failure | 2.61 (2.21 ; 3.08) | **<0.001** | 1.000 (0.67 ; 1.49) | >0.99 |
| Myocardial infarction | 2.15 (1.57 ; 2.94) | **<0.001** | 1.25 (0.77 ; 2.01) | 0.37 |
| Cardiac arrythmia | 2.15 (1.82 ; 2.53) | **<0.001** | 1.09 (0.75 ; 1.60) | 0.65 |
| Valvular disease | 2.18 (1.59 ; 2.99) | **<0.001** | 1.34 (0.83 ; 2.17) | 0.23 |
| Peripheral vascular disease | 2.13 (1.66 ; 2.74) | **<0.001** | 0.93 (0.60 ; 1.43) | 0.73 |
| Coagulopathy | 2.70 (2.29 ; 3.18) | **<0.001** | 1.25 (0.86 ; 1.84) | 0.25 |
| Rheumatoid arthritis/collagen vas | 1.32 (0.997 ; 1.75) | 0.052 | 1.10 (0.70 ; 1.72) | 0.68 |
| Organ transplant | 2.47 (2.07 ; 2.96) | **<0.001** | 1.25 (0.84 ; 1.86) | 0.27 |
| Chronic pulmonary disease | 1.22 (1.01 ; 1.47) | **0.039** | 1.006 (0.68 ; 1.49) | 0.98 |
| Pulmonary circulation disease | 2.60 (2.21 ; 3.05) | **<0.001** | 1.30 (0.89 ; 1.89) | 0.17 |
| Renal failure | 2.95 (2.54 ; 3.43) | **<0.001** | 1.34 (0.90 ; 1.99) | 0.15 |
| Liver disease | 2.52 (2.16 ; 2.93) | **<0.001** | 1.33 (0.90 ; 1.96) | 0.16 |
| Cerebrovascular disease | 2.56 (2.12 ; 3.10) | **<0.001** | 1.59 (1.06 ; 2.39) | **0.025** |
| Paralysis | 1.12 (0.85 ; 1.47) | 0.42 | 0.56 (0.36 ; 0.88) | **0.011** |
| Dementia | 1.68 (1.06 ; 2.68) | **0.028** | 1.04 (0.57 ; 1.90) | 0.90 |
| Other neurological disorders | 1.73 (1.42 ; 2.12) | **<0.001** | 1.11 (0.74 ; 1.67) | 0.60 |
| Solid tumor without metastasis | 3.03 (2.57 ; 3.59) | **<0.001** | 1.40 (0.93 ; 2.11) | 0.10 |
| Metastatic cancer | 4.09 (3.35 ; 5.00) | **<0.001** | 2.63 (1.68 ; 4.11) | **<0.001** |
| Lymphoma | 2.54 (1.92 ; 3.38) | **<0.001** | 1.39 (0.87 ; 2.24) | 0.17 |
| Acquired immune deficiency syndrome | 1.47 (1.07 ; 2.02) | **0.018** |  |  |
| Na, mmol/L | 0.95 (0.93 ; 0.96) | **<0.001** | 0.998 (0.99 ; 1.01) | 0.75 |
| K, mmol/L | 1.72 (1.57 ; 1.88) | **<0.001** | 1.10 (1.000 ; 1.20) | 0.050 |
| Hemoglobin, g/dL | 0.85 (0.83 ; 0.87) | **<0.001** | 0.93 (0.90 ; 0.96) | **<0.001** |
| Leukocyte count, x 10^9 / L | 1.05 (1.04 ; 1.05) | **<0.001** | 0.98 (0.94 ; 1.02) | 0.29 |
| Lymphocyte count, x 10^9 / L | 0.65 (0.58 ; 0.72) | **<0.001** | 1.02 (0.96 ; 1.09) | 0.50 |
| Neutrophil count, x 10^9 / L | 1.07 (1.06 ; 1.08) | **<0.001** | 1.04 (1.000 ; 1.09) | **0.048** |
| Eosinophil count, x 10^9 / L | 0.01 (0.00 ; 0.02) | **<0.001** | 0.18 (0.07 ; 0.51) | **0.001** |
| Platelet count, x 10^9 / L | 0.997 (0.997 ; 0.998) | **<0.001** | 0.998 (0.997 ; 0.999) | **<0.001** |
| Serum creatinine, µmol/L | 1.002 (1.002 ; 1.002) | **<0.001** | 0.999 (0.999 ; 1.000) | **0.020** |
| Urea, mmol/L | 1.06 (1.06 ; 1.07) | **<0.001** | 1.03 (1.02 ; 1.04) | **<0.001** |
| C-reactive protein (mg/L) | 1.005 (1.004 ; 1.005) | **<0.001** | 1.001 (1.000 ; 1.002) | **0.015** |
| Protides, g/L | 0.94 (0.93 ; 0.95) | **<0.001** | 0.98 (0.97 ; 0.99) | **<0.001** |
| Alanine transaminase, U/L | 1.003 (1.002 ; 1.004) | **<0.001** | 0.998 (0.996 ; 0.999) | **<0.001** |
| Bilirubin total, µmol/L | 1.007 (1.006 ; 1.009) | **<0.001** | 1.005 (1.003 ; 1.007) | **<0.001** |
| Partial thromboplastin time (PTT) | 1.95 (1.80 ; 2.10) | **<0.001** | 1.15 (1.02 ; 1.30) | **0.026** |
| Prothrombin time (%) | 0.97 (0.97 ; 0.97) | **<0.001** | 0.998 (0.994 ; 1.003) | 0.45 |
| Glycemia, mmol/L | 1.13 (1.11 ; 1.14) | **<0.001** | 1.05 (1.03 ; 1.07) | **<0.001** |
| HCO3- serum, mmol/L | 0.88 (0.87 ; 0.90) | **<0.001** | 0.94 (0.92 ; 0.96) | **<0.001** |
| Calcium, mmol/L | 0.06 (0.05 ; 0.07) | **<0.001** | 0.50 (0.34 ; 0.72) | **<0.001** |
| Fibrinogen, g/L | 1.17 (1.13 ; 1.21) | **<0.001** | 1.003 (0.96 ; 1.05) | 0.90 |
| Procalcitonin, µg/L | 1.08 (1.07 ; 1.10) | **<0.001** | 0.98 (0.96 ; 0.998) | **0.028** |
| Troponin, ng/L | 1.001 (1.000 ; 1.001) | **<0.001** | 1.000 (1.000 ; 1.000) | 0.68 |
| Lactate dehydrogenase, U/L | 1.003 (1.003 ; 1.003) | **<0.001** | 1.002 (1.002 ; 1.002) | **<0.001** |
| Creatine kinase, U/L | 1.000 (1.000 ; 1.000) | **<0.001** | 1.000 (1.000 ; 1.000) | 0.16 |
| D-Dimer (µg/L) | 1.000 (1.000 ; 1.000) | **<0.001** | 1.000 (1.000 ; 1.000) | **0.035** |
| Arterial blood gas test: SpO2, % | 0.98 (0.97 ; 0.98) | **<0.001** | 0.99 (0.98 ; 0.996) | **0.004** |
| Arterial blood gas test: PaO2, mmHg | 1.003 (1.002 ; 1.005) | **<0.001** | 1.001 (0.999 ; 1.002) | 0.32 |
| Arterial blood gas test: PaCO2, mmHg | 1.006 (0.999 ; 1.01) | 0.078 | 0.98 (0.98 ; 0.991) | **<0.001** |
| Arterial blood gas test: HCO3-, mmol/L | 0.92 (0.91 ; 0.94) | **<0.001** | 1.09 (1.06 ; 1.12) | **<0.001** |
| Arterial blood gas test: lactate, mmol/L | 1.27 (1.24 ; 1.30) | **<0.001** | 0.999 (0.95 ; 1.05) | 0.98 |
| Arterial blood gas test: pH | 0.00 (0.00 ; 0.01) | **<0.001** | 0.04 (0.01 ; 0.11) | **<0.001** |
| ^1^Hazard ratio (95% confidence interval) from unadjusted Cox proportional-hazards regression modeling. | | | | |
| ^2^Hazard ratio (95% confidence interval) from multivariable Cox proportional-hazards regression modeling. | | | | |

**Supplemental Table 6**. Results from univariate and multivariate Cox proportional hazards modeling for 90-day overall survival: patients ≥ 65 years old

|  | Univariable analysis | | Multivariable analysis | |  |
| --- | --- | --- | --- | --- | --- |
| **Characteristic** | **HR (95% CI)**^1^ | **p-value** | **HR (95% CI)**^2^ | **p-value** |  |
| Parental status |  |  |  |  |  |
| Without children | — |  | — |  |  |
| With children | 1.03 (0.95 ; 1.11) | 0.49 | 1.07 (0.990 ; 1.15) | 0.088 |  |
| Period (COVID-19 wave) |  | **<0.001** |  |  |  |
| 1 | — |  | — |  |  |
| 2 | 0.75 (0.69 ; 0.81) | **<0.001** | 0.78 (0.71 ; 0.85) | **<0.001** |  |
| 3 | 0.85 (0.79 ; 0.91) | **<0.001** | 0.86 (0.80 ; 0.93) | **<0.001** |  |
| 4 | 0.82 (0.71 ; 0.94) | **0.004** | 0.81 (0.71 ; 0.94) | **0.004** |  |
| 5 | 0.72 (0.66 ; 0.79) | **<0.001** | 0.72 (0.66 ; 0.79) | **<0.001** |  |
| Age, years | 1.03 (1.03 ; 1.04) | **<0.001** | 1.05 (1.05 ; 1.06) | **<0.001** |  |
| Male sex | 1.32 (1.24 ; 1.40) | **<0.001** | 1.25 (1.18 ; 1.34) | **<0.001** |  |
| Number of comorbidities | 1.002 (0.990 ; 1.01) | 0.79 | 0.73 (0.49 ; 1.09) | 0.12 |  |
| Diabetes | 0.89 (0.84 ; 0.95) | **<0.001** | 1.16 (0.77 ; 1.73) | 0.47 |  |
| Obesity | 0.84 (0.77 ; 0.93) | **<0.001** | 1.28 (0.85 ; 1.93) | 0.23 |  |
| Body mass index, kg/m² | 0.996 (0.99 ; 1.004) | 0.30 | 1.02 (1.009 ; 1.03) | **<0.001** |  |
| Hypertension | 0.86 (0.81 ; 0.91) | **<0.001** | 1.16 (0.78 ; 1.73) | 0.47 |  |
| Congestive heart failure | 1.18 (1.11 ; 1.26) | **<0.001** | 1.40 (0.94 ; 2.11) | 0.10 |  |
| Myocardial infarction | 1.003 (0.88 ; 1.14) | 0.97 | 1.33 (0.88 ; 2.03) | 0.18 |  |
| Cardiac arrythmia | 1.15 (1.08 ; 1.22) | **<0.001** | 1.34 (0.89 ; 2.00) | 0.16 |  |
| Valvular disease | 1.02 (0.91 ; 1.14) | 0.71 | 1.28 (0.85 ; 1.93) | 0.24 |  |
| Peripheral vascular disease | 0.998 (0.91 ; 1.10) | 0.97 | 1.30 (0.86 ; 1.95) | 0.21 |  |
| Coagulopathy | 1.17 (1.06 ; 1.29) | **0.002** | 1.24 (0.82 ; 1.87) | 0.31 |  |
| Rheumatoid arthritis/collagen vas | 0.94 (0.80 ; 1.10) | 0.43 | 1.61 (1.05 ; 2.47) | **0.029** |  |
| Organ transplant | 1.22 (1.04 ; 1.43) | **0.014** | 1.98 (1.28 ; 3.05) | **0.002** |  |
| Chronic pulmonary disease | 0.95 (0.87 ; 1.02) | 0.17 | 1.41 (0.94 ; 2.11) | 0.10 |  |
| Pulmonary circulation disease | 0.992 (0.90 ; 1.09) | 0.86 | 1.32 (0.88 ; 1.98) | 0.18 |  |
| Renal failure | 1.15 (1.08 ; 1.23) | **<0.001** | 1.37 (0.91 ; 2.06) | 0.13 |  |
| Liver disease | 0.91 (0.81 ; 1.04) | 0.16 | 1.29 (0.84 ; 1.96) | 0.24 |  |
| Cerebrovascular disease | 0.87 (0.80 ; 0.94) | **<0.001** | 1.20 (0.80 ; 1.80) | 0.39 |  |
| Paralysis | 0.87 (0.77 ; 0.99) | **0.030** | 1.42 (0.93 ; 2.15) | 0.10 |  |
| Dementia | 0.96 (0.89 ; 1.03) | 0.21 | 1.19 (0.80 ; 1.79) | 0.40 |  |
| Other neurological disorders | 0.996 (0.92 ; 1.08) | 0.92 | 1.65 (1.10 ; 2.48) | **0.015** |  |
| Solid tumor without metastasis | 1.05 (0.97 ; 1.14) | 0.24 | 1.29 (0.86 ; 1.94) | 0.23 |  |
| Metastatic cancer | 1.39 (1.25 ; 1.56) | **<0.001** | 2.32 (1.52 ; 3.53) | **<0.001** |  |
| Lymphoma | 1.29 (1.12 ; 1.48) | **<0.001** | 1.78 (1.17 ; 2.72) | **0.007** |  |
| Acquired immune deficiency syndrome | 0.62 (0.42 ; 0.92) | **0.017** |  |  |  |
| Na, mmol/L | 1.02 (1.01 ; 1.02) | **<0.001** | 1.02 (1.010 ; 1.02) | **<0.001** |  |
| K, mmol/L | 1.20 (1.15 ; 1.26) | **<0.001** | 1.08 (1.03 ; 1.13) | **0.002** |  |
| Hemoglobin, g/dL | 0.97 (0.96 ; 0.99) | **<0.001** | 0.99 (0.97 ; 1.005) | 0.18 |  |
| Leukocyte count, x 10^9 / L | 1.03 (1.02 ; 1.03) | **<0.001** | 0.99 (0.97 ; 1.002) | 0.091 |  |
| Lymphocyte count, x 10^9 / L | 0.98 (0.96 ; 1.003) | 0.10 | 1.01 (0.99 ; 1.04) | 0.30 |  |
| Neutrophil count, x 10^9 / L | 1.05 (1.04 ; 1.05) | **<0.001** | 1.02 (1.004 ; 1.04) | **0.014** |  |
| Eosinophil count, x 10^9 / L | 0.02 (0.01 ; 0.04) | **<0.001** | 0.19 (0.11 ; 0.33) | **<0.001** |  |
| Platelet count, x 10^9 / L | 0.998 (0.997 ; 0.998) | **<0.001** | 0.998 (0.998 ; 0.999) | **<0.001** |  |
| Serum creatinine, µmol/L | 1.001 (1.001 ; 1.001) | **<0.001** | 0.999 (0.999 ; 1.000) | **<0.001** |  |
| Urea, mmol/L | 1.03 (1.03 ; 1.04) | **<0.001** | 1.02 (1.01 ; 1.03) | **<0.001** |  |
| C-reactive protein (mg/L) | 1.004 (1.004 ; 1.004) | **<0.001** | 1.003 (1.003 ; 1.004) | **<0.001** |  |
| Protides, g/L | 0.98 (0.98 ; 0.99) | **<0.001** | 0.992 (0.99 ; 0.996) | **<0.001** |  |
| Alanine transaminase, U/L | 1.001 (1.001 ; 1.002) | **<0.001** | 0.998 (0.997 ; 0.998) | **<0.001** |  |
| Bilirubin total, µmol/L | 1.006 (1.005 ; 1.008) | **<0.001** | 1.003 (1.001 ; 1.005) | **0.006** |  |
| Partial thromboplastin time (PTT) | 1.48 (1.39 ; 1.56) | **<0.001** | 1.17 (1.08 ; 1.26) | **<0.001** |  |
| Prothrombin time (%) | 0.99 (0.99 ; 0.99) | **<0.001** | 0.997 (0.995 ; 0.999) | **0.003** |  |
| Glycemia, mmol/L | 1.07 (1.06 ; 1.08) | **<0.001** | 1.04 (1.03 ; 1.05) | **<0.001** |  |
| HCO3- serum, mmol/L | 0.93 (0.92 ; 0.93) | **<0.001** | 0.97 (0.96 ; 0.98) | **<0.001** |  |
| Calcium, mmol/L | 0.23 (0.19 ; 0.27) | **<0.001** | 0.54 (0.44 ; 0.67) | **<0.001** |  |
| Fibrinogen, g/L | 1.07 (1.05 ; 1.09) | **<0.001** | 0.91 (0.89 ; 0.94) | **<0.001** |  |
| Procalcitonin, µg/L | 1.06 (1.05 ; 1.06) | **<0.001** | 0.996 (0.99 ; 1.006) | 0.43 |  |
| Troponin, ng/L | 1.000 (1.000 ; 1.000) | **<0.001** | 1.000 (1.000 ; 1.000) | **<0.001** |  |
| Lactate dehydrogenase, U/L | 1.002 (1.002 ; 1.002) | **<0.001** | 1.002 (1.002 ; 1.002) | **<0.001** |  |
| Creatine kinase, U/L | 1.000 (1.000 ; 1.000) | **<0.001** | 1.000 (1.000 ; 1.000) | **0.004** |  |
| D-Dimer (µg/L) | 1.000 (1.000 ; 1.000) | **<0.001** | 1.000 (1.000 ; 1.000) | **0.003** |  |
| Arterial blood gas test: SpO2, % | 0.97 (0.97 ; 0.97) | **<0.001** | 0.99 (0.99 ; 0.993) | **<0.001** |  |
| Arterial blood gas test: PaO2, mmHg | 0.999 (0.998 ; 1.000) | 0.12 | 0.999 (0.998 ; 1.000) | **0.019** |  |
| Arterial blood gas test: PaCO2, mmHg | 0.994 (0.991 ; 0.998) | **<0.001** | 0.997 (0.993 ; 1.001) | 0.17 |  |
| Arterial blood gas test: HCO3-, mmol/L | 0.93 (0.92 ; 0.94) | **<0.001** | 1.03 (1.02 ; 1.05) | **<0.001** |  |
| Arterial blood gas test: lactate, mmol/L | 1.23 (1.21 ; 1.24) | **<0.001** | 1.06 (1.04 ; 1.09) | **<0.001** |  |
| Arterial blood gas test: pH | 0.02 (0.01 ; 0.02) | **<0.001** | 0.06 (0.03 ; 0.12) | **<0.001** |  |
| ^1^Hazard ratio (95% confidence interval) from unadjusted Cox proportional-hazards regression modeling. | | | | | |
| ^2^Hazard ratio (95% confidence interval) from multivariable Cox proportional-hazards regression modeling. | | | | | |

**Supplemental** **Table 7**. Main characteristics of the study population according to parental status (3 categories)

| **Characteristic** | **Overall**  N = 30386^1^ | **Explicitly Without children**  N=3567^1^ | **Explicitly With children**  N=22713^1^ | **No mention**  N=4106^1^ | **p-value**^2^ |
| --- | --- | --- | --- | --- | --- |
| ***Demographics*** |  |  |  |  |  |
| Age, years | 63.7 ± 19.1 | 63.5 ± 20.3 | 66.2 ± 17.5 | 50.1 ± 20.4 | **<0.001** |
| Male sex | 16,801 (55.3%) | 2,042 (57.2%) | 12,359 (54.4%) | 2,400 (58.5%) | **<0.001** |
| ***Outcomes*** |  |  |  |  |  |
| 28-day mortality | 4,572 (15.0%) | 503 (14.1%) | 3,680 (16.2%) | 389 (9.5%) | **<0.001** |
| 90-day mortality | 5,711 (18.8%) | 663 (18.6%) | 4,559 (20.1%) | 489 (11.9%) | **<0.001** |
| In hospital mortality | 4,917 (16.2%) | 539 (15.1%) | 3,922 (17.3%) | 456 (11.1%) | **<0.001** |
| ICU transfer | 8,960 (29.5%) | 1,040 (29.2%) | 6,845 (30.1%) | 1,075 (26.2%) | **<0.001** |
| Length of stay, days | 9.0 [5.0;16.0] | 11.0 [6.0;18.0] | 9.0 [5.0;17.0] | 6.0 [3.00;11.0] | **<0.001** |
| ***Comorbidities*** |  |  |  |  |  |
| Number of comorbidities | 2.00 [1.00;4.00] | 2.00 [1.00;4.00] | 2.00 [1.00;4.00] | 1.00 [0.000;2.00] | **<0.001** |
| Diabetes | 8,199 (27.0%) | 828 (23.2%) | 6,853 (30.2%) | 518 (12.6%) | **<0.001** |
| Obesity | 4,876 (16.0%) | 575 (16.1%) | 3,863 (17.0%) | 438 (10.7%) | **<0.001** |
| Body mass index (BMI), kg/m² | 27.2 ± 4.23 | 26.9 ± 4.62 | 27.3 ± 4.21 | 27.1 ± 3.96 | **<0.001** |
| Hypertension | 12,986 (42.7%) | 1,519 (42.6%) | 10,670 (47.0%) | 797 (19.4%) | **<0.001** |
| Congestive heart failure | 5,073 (16.7%) | 655 (18.4%) | 4,188 (18.4%) | 230 (5.6%) | **<0.001** |
| Cardiac arrythmia | 6,024 (19.8%) | 766 (21.5%) | 4,893 (21.5%) | 365 (8.9%) | **<0.001** |
| Chronic pulmonary disease | 3,927 (12.9%) | 514 (14.4%) | 3,094 (13.6%) | 319 (7.8%) | **<0.001** |
| Renal failure | 4,815 (15.8%) | 668 (18.7%) | 3,970 (17.5%) | 177 (4.3%) | **<0.001** |
| Dementia | 3,944 (13.0%) | 616 (17.3%) | 3,169 (14.0%) | 159 (3.9%) | **<0.001** |
| Solid tumor w/out metastasis | 3,085 (10.2%) | 420 (11.8%) | 2,473 (10.9%) | 192 (4.7%) | **<0.001** |
| Metastatic cancer | 1,256 (4.1%) | 176 (4.9%) | 1,021 (4.5%) | 59 (1.4%) | **<0.001** |
| ***Biological parameters*** |  |  |  |  |  |
| Na, mmol/L | 137 [134;139] | 137 [134;139] | 136 [134;139] | 137 [135;139] | **<0.001** |
| K, mmol/L | 4.06 [3.78;4.40] | 4.03 [3.72;4.38] | 4.09 [3.79;4.40] | 4.00 [3.80;4.30] | **<0.001** |
| Hemoglobin, g/dL | 12.8 [11.6;14.1] | 13.0 [11.8;14.2] | 12.8 [11.6;14.0] | 13.1 [11.8;14.4] | **<0.001** |
| Leukocytes count, x 10^9 / L | 7.0 [5.2;9.2] | 7.0 [5.2;9.3] | 7.0 [5.2;9.1] | 7.6 [5.7;10.0] | **<0.001** |
| Lymphocyte count, x 10^9 / L | 1.03 [0.70;1.41] | 1.00 [0.68;1.38] | 1.01 [0.70;1.38] | 1.14 [0.78;1.63] | **<0.001** |
| Platelet count, x 10^9 / L | 219 [170;269] | 221 [170;275] | 217 [168;267] | 227 [180;274] | **<0.001** |
| Serum creatinine, µmol/L | 79.0 [62.0;107] | 80.0 [64.0;107] | 81.0 [63.0;109] | 72.4 [57.2;92.0] | **<0.001** |
| Urea, mmol/L | 6.1 [4.30;9.3] | 6.3 [4.50;9.9] | 6.3 [4.50;9.6] | 4.80 [3.40;7.1] | **<0.001** |
| C reactive protein (mg/L) | 59.8 [20.0;121] | 57.0 [19.1;115] | 63.0 [22.6;125] | 42.0 [11.7;105] | **<0.001** |
| Protides, g/L | 71.0 [66.8;75.0] | 71.0 [66.9;75.6] | 71.0 [66.3;75.0] | 72.0 [68.0;75.5] | **<0.001** |
| Lacticodeshydrogenase, U/L | 363 [280;474] | 368 [284;481] | 366 [283;474] | 337 [254;462] | **<0.001** |
| D-Dimer (µg/L) | 1107 [744;1674] | 1137 [768;1722] | 1113 [756;1674] | 1043 [678;1625] | **<0.001** |
| BGA: Oxygen saturation, % | 94.4 [92.3;95.8] | 94.4 [92.3;95.8] | 94.4 [92.2;95.8] | 94.7 [92.8;96.0] | **<0.001** |
| BGA: PaO2, mmHg | 73.3 [63.1;81.3] | 74.0 [64.0;81.5] | 73.1 [62.7;81.0] | 74.3 [65.0;83.1] | **<0.001** |
| BGA: PaCO2, mmHg | 35.2 [32.1;38.1] | 35.0 [32.0;37.8] | 35.2 [32.0;38.1] | 35.4 [32.5;38.3] | **0.001** |
| ^1^Mean ± SD; n (%); Median [Q1;Q3] | | | | | |
| ^2^One-way analysis of means; Pearson's Chi-squared test; Kruskal-Wallis rank sum test | | | | | |

**Supplemental Table 8.** Age-standardized main baseline characteristics of the study population, stratified by parental status.

| **Characteristic** | **Explicitly without children N=3567** | **Explicitly with children N=22713** | **No mention N=4106** |
| --- | --- | --- | --- |
| 28-day mortality | 14.2% | 15.1% | 16.5% |
| 90-day mortality | 18.8% | 18.7% | 19.8% |
| In hospital mortality | 15.4% | 16.1% | 18.2% |
| ICU transfer | 30.5% | 29.8% | 27.5% |
| Length of stay, days | 15.34 (0.30) | 13.71 (0.11) | 11.42 (0.24) |
| Male sex | 58.2% | 52.9% | 63.8% |
| Diabetes | 24.1% | 28.8% | 16.5% |
| Obesity | 16.5% | 17.1% | 9.9% |
| Hypertension | 43.2% | 44.3% | 29.6% |
| Congestive heart failure | 18.2% | 17.3% | 9.2% |
| Cardiac arrythmia | 21.2% | 20.2% | 13.5% |
| Chronic pulmonary disease | 14.8% | 13.1% | 9.7% |
| Renal failure | 18.3% | 16.5% | 6.4% |
| Dementia | 16.2% | 12.8% | 8.4% |
| Solid tumor w/out metastasis | 12.1% | 10.3% | 6.8% |
| Metastatic cancer | 5.2% | 4.3% | 2.0% |
| Body mass index (BMI), kg/m² | 26.98 (0.075) | 27.31 (0.028) | 26.96 (0.063) |
| Number of comorbidities | 2.77 (0.038) | 2.65 (0.015) | 1.55 (0.033) |
| Na, mmol/L | 136.28 (0.082) | 136.32 (0.029) | 136.77 (0.102) |
| K, mmol/L | 4.08 (0.0102) | 4.12 (0.0039) | 4.09 (0.0113) |
| Hemoglobin, g/dL | 12.89 (0.035) | 12.66 (0.013) | 13.11 (0.038) |
| Leukocytes count, x 10^9 / L | 7.89 (0.079) | 7.80 (0.030) | 8.24 (0.083) |
| Lymphocyte count, x 10^9 / L | 1.20 (0.032) | 1.21 (0.010) | 1.15 (0.019) |
| Platelet count, x 10^9 / L | 233.41 (1.68) | 227.93 (0.63) | 233.74 (1.75) |
| Serum creatinine, µmol/L | 107.58 (1.98) | 106.08 (0.71) | 97.25 (1.53) |
| Urea, mmol/L | 8.69 (0.125) | 8.06 (0.041) | 8.01 (0.129) |
| C reactive protein (mg/L) | 82.33 (1.33) | 83.47 (0.52) | 84.74 (1.51) |
| Protides, g/L | 70.92 (0.13) | 70.84 (0.05) | 70.98 (0.14) |
| Lacticodeshydrogenase, U/L | 410.63 (3.02) | 399.27 (1.16) | 410.59 (3.29) |
| D-Dimer (µg/L) | 1674.27 (31.06) | 1587.67 (11.50) | 1775.78 (39.82) |
| BGA: Oxygen saturation, % | 93.23 (0.094) | 92.98 (0.045) | 93.30 (0.119) |
| BGA: PaO2, mmHg | 73.13 (0.56) | 72.72 (0.21) | 75.16 (0.66) |
| BGA: PaCO2, mmHg | 33.96 (0.15) | 34.29 (0.06) | 34.38 (0.17) |

Results are percentages (%) or means (standard error), adjusted by age via direct standardization.

**Supplemental Table 9**. Results from univariate and multivariable Cox proportional hazards modeling for 90-day overall survival: results according to parental status as a 3-categories variable

|  | Univariate analysis | | Multivariable analysis | | | | |
| --- | --- | --- | --- | --- | --- | --- | --- |
| **Characteristic** | **HR (95% CI)**^1^ | **p-value** | **HR (95% CI)**^2^ | **p-value** | | | |
| ***Whole study population*** |  |  |  |  | | | |
| Parental status |  | **<0.001** |  |  | | | |
| Explicitly without children | — |  | — |  | | | |
| Explicitly with children | 1.09 (1.009 ; 1.19) | **0.029** | 1.02 (0.94 ; 1.11) | 0.61 | | | |
| No mention | 0.62 (0.55 ; 0.70) | **<0.001** | 1.04 (0.93 ; 1.18) | 0.47 | | | |
| ***In patients aged <65 years old*** |  |  |  |  | | | |
| Parental status |  | **<0.001** |  |  | | | |
| Explicitly without children | — |  | — |  | | | |
| Explicitly with children | 0.97 (0.82 ; 1.16) | 0.77 | 0.81 (0.68 ; 0.97) | **0.024** | | | |
| No mention | 0.63 (0.50 ; 0.79) | **<0.001** | 0.95 (0.76 ; 1.20) | 0.68 | | | |
| ***In patients aged >65 years old*** | | | |  |  |  |  |
| Parental status |  | 0.75 |  |  | | | |
| Explicitly without children | — |  | — |  | | | |
| Explicitly with children | 1.04 (0.94 ; 1.13) | 0.46 | 1.10 (0.998 ; 1.20) | 0.054 | | | |
| No mention | 1.02 (0.89 ; 1.17) | 0.75 | 1.07 (0.93 ; 1.24) | 0.32 | | | |
| ^1^Hazard ratio (95% confidence interval) from unadjusted Cox proportional hazards regression modeling. | | | | | | | |
| ^2^Hazard ratio (95% confidence interval) from multivariable Cox proportional hazards regression modeling. | | | | | | | |
| Abbreviations: CI = Confidence Interval, HR = Hazard Ratio | | | | | | | |

**Supplemental Table 10.** Sequential multivariable Cox proportional hazards modeling for 90-day overall survival: whole population

|  | **Model #1** | | **Model #2** | | **Model #3** | |
| --- | --- | --- | --- | --- | --- | --- |
| **Characteristic** | **HR (95% CI)**^1^ | **p-value** | **HR (95% CI)**^1^ | **p-value** | **HR (95% CI)**^1^ | **p-value** |
| Parental status |  |  |  |  |  |  |
| Without children | — |  | — |  | — |  |
| With children | 1.04 (0.97 ; 1.11) | 0.27 | 0.999 (0.94 ; 1.07) | 0.98 | 1.003 (0.94 ; 1.07) | 0.92 |
| Period (COVID-19 wave) |  |  |  |  |  |  |
| 1 | — |  | — |  | — |  |
| 2 | 0.76 (0.70 ; 0.82) | **<0.001** | 0.74 (0.69 ; 0.80) | **<0.001** | 0.78 (0.72 ; 0.84) | **<0.001** |
| 3 | 0.86 (0.81 ; 0.92) | **<0.001** | 0.84 (0.79 ; 0.90) | **<0.001** | 0.85 (0.79 ; 0.91) | **<0.001** |
| 4 | 0.85 (0.75 ; 0.96) | **0.010** | 0.82 (0.73 ; 0.93) | **0.002** | 0.82 (0.73 ; 0.93) | **0.002** |
| 5 | 0.73 (0.68 ; 0.79) | **<0.001** | 0.70 (0.65 ; 0.76) | **<0.001** | 0.75 (0.70 ; 0.82) | **<0.001** |
| Age, years | 1.05 (1.05 ; 1.05) | **<0.001** | 1.06 (1.06 ; 1.06) | **<0.001** | 1.06 (1.06 ; 1.06) | **<0.001** |
| Male sex | 1.61 (1.52 ; 1.70) | **<0.001** | 1.55 (1.47 ; 1.64) | **<0.001** | 1.27 (1.20 ; 1.35) | **<0.001** |
| Number of comorbidities |  |  | 0.96 (0.75 ; 1.24) | 0.78 | 0.90 (0.70 ; 1.16) | 0.43 |
| Diabetes |  |  | 1.02 (0.79 ; 1.32) | 0.89 | 0.94 (0.73 ; 1.22) | 0.65 |
| Obesity |  |  | 1.03 (0.79 ; 1.34) | 0.83 | 1.06 (0.81 ; 1.38) | 0.68 |
| Body mass index (BMI), kg/m² |  |  | 1.03 (1.02 ; 1.04) | **<0.001** | 1.02 (1.01 ; 1.03) | **<0.001** |
| Hypertension |  |  | 0.85 (0.66 ; 1.10) | 0.22 | 0.94 (0.73 ; 1.21) | 0.63 |
| Congestive heart failure |  |  | 1.13 (0.87 ; 1.47) | 0.35 | 1.13 (0.87 ; 1.47) | 0.35 |
| Myocardial infarction |  |  | 1.03 (0.78 ; 1.35) | 0.86 | 1.10 (0.83 ; 1.45) | 0.52 |
| Cardiac arrythmia |  |  | 1.04 (0.80 ; 1.34) | 0.79 | 1.10 (0.85 ; 1.42) | 0.48 |
| Valvular disease |  |  | 0.95 (0.72 ; 1.25) | 0.71 | 1.03 (0.79 ; 1.36) | 0.81 |
| Peripheral vascular disease |  |  | 0.98 (0.75 ; 1.28) | 0.88 | 1.04 (0.80 ; 1.36) | 0.77 |
| Coagulopathy |  |  | 1.31 (1.005 ; 1.71) | **0.046** | 1.09 (0.84 ; 1.42) | 0.52 |
| Rheumatoid arthritis/collagen vas |  |  | 1.16 (0.87 ; 1.54) | 0.31 | 1.33 (0.999 ; 1.77) | 0.050 |
| Organ transplant |  |  | 1.80 (1.36 ; 2.38) | **<0.001** | 1.72 (1.30 ; 2.28) | **<0.001** |
| Chronic pulmonary disease |  |  | 0.99 (0.76 ; 1.28) | 0.94 | 1.15 (0.89 ; 1.50) | 0.29 |
| Pulmonary circulation disease |  |  | 1.18 (0.91 ; 1.53) | 0.22 | 1.15 (0.89 ; 1.50) | 0.29 |
| Renal failure |  |  | 1.15 (0.89 ; 1.49) | 0.29 | 1.12 (0.86 ; 1.46) | 0.39 |
| Liver disease |  |  | 1.26 (0.96 ; 1.65) | 0.10 | 1.23 (0.93 ; 1.62) | 0.14 |
| Cerebrovascular disease |  |  | 0.94 (0.72 ; 1.23) | 0.66 | 1.05 (0.81 ; 1.38) | 0.70 |
| Paralysis |  |  | 0.97 (0.74 ; 1.28) | 0.85 | 1.03 (0.78 ; 1.36) | 0.82 |
| Dementia |  |  | 0.74 (0.57 ; 0.96) | **0.026** | 0.96 (0.74 ; 1.25) | 0.77 |
| Other neurological disorders |  |  | 1.14 (0.88 ; 1.48) | 0.32 | 1.34 (1.03 ; 1.75) | **0.028** |
| Solid tumor w/out metastasis |  |  | 0.97 (0.74 ; 1.26) | 0.82 | 1.08 (0.83 ; 1.41) | 0.56 |
| Metastatic cancer |  |  | 1.86 (1.41 ; 2.45) | **<0.001** | 2.09 (1.58 ; 2.76) | **<0.001** |
| Lymphoma |  |  | 1.28 (0.97 ; 1.70) | 0.084 | 1.45 (1.09 ; 1.93) | **0.010** |
| Na, mmol/L |  |  |  |  | 1.01 (1.006 ; 1.02) | **<0.001** |
| K, mmol/L |  |  |  |  | 1.08 (1.04 ; 1.13) | **<0.001** |
| Hemoglobin, g/dL |  |  |  |  | 0.97 (0.96 ; 0.99) | **<0.001** |
| Leukocytes count, x 10^9 / L |  |  |  |  | 0.99 (0.97 ; 1.004) | 0.15 |
| Lymphocyte count, x 10^9 / L |  |  |  |  | 1.006 (0.99 ; 1.03) | 0.56 |
| Neutrophil count, x 10^9 / L |  |  |  |  | 1.02 (1.007 ; 1.04) | **0.006** |
| Eosinophil count, x 10^9 / L |  |  |  |  | 0.19 (0.12 ; 0.31) | **<0.001** |
| Platelet count, x 10^9 / L |  |  |  |  | 0.998 (0.998 ; 0.999) | **<0.001** |
| Serum creatinine, µmol/L |  |  |  |  | 0.999 (0.999 ; 1.000) | **<0.001** |
| Urea, mmol/L |  |  |  |  | 1.02 (1.02 ; 1.03) | **<0.001** |
| C reactive protein (mg/L) |  |  |  |  | 1.003 (1.002 ; 1.003) | **<0.001** |
| Protides, g/L |  |  |  |  | 0.99 (0.99 ; 0.994) | **<0.001** |
| ALT, U/L |  |  |  |  | 0.997 (0.997 ; 0.998) | **<0.001** |
| Bilirubin total, µmol/L |  |  |  |  | 1.004 (1.003 ; 1.006) | **<0.001** |
| Partial thromboplastin time (PTT) |  |  |  |  | 1.16 (1.08 ; 1.23) | **<0.001** |
| Prothrombin time (%) |  |  |  |  | 0.997 (0.996 ; 0.999) | **0.003** |
| Glycemia, mmol/L |  |  |  |  | 1.04 (1.03 ; 1.05) | **<0.001** |
| HCO3- serum, mmol/L |  |  |  |  | 0.96 (0.95 ; 0.97) | **<0.001** |
| Calcium, mmol/L |  |  |  |  | 0.51 (0.42 ; 0.61) | **<0.001** |
| Fibrinogen, g/L |  |  |  |  | 0.94 (0.92 ; 0.96) | **<0.001** |
| Procalcitonin, µg/L |  |  |  |  | 0.993 (0.99 ; 1.002) | 0.12 |
| Troponin, ng/L |  |  |  |  | 1.000 (1.000 ; 1.000) | **<0.001** |
| Lacticodeshydrogenase, U/L |  |  |  |  | 1.002 (1.002 ; 1.002) | **<0.001** |
| Creatine kinase, U/L |  |  |  |  | 1.000 (1.000 ; 1.000) | **0.016** |
| D-Dimer (µg/L) |  |  |  |  | 1.000 (1.000 ; 1.000) | 0.090 |
| BGA: Oxygen saturation, % |  |  |  |  | 0.99 (0.99 ; 0.992) | **<0.001** |
| BGA: PaO2, mmHg |  |  |  |  | 0.999 (0.998 ; 1.000) | 0.13 |
| BGA: PaCO2, mmHg |  |  |  |  | 0.994 (0.990 ; 0.998) | **0.001** |
| BGA: HCO3-, mmol/L |  |  |  |  | 1.04 (1.03 ; 1.06) | **<0.001** |
| BGA: lactate, mmol/L |  |  |  |  | 1.05 (1.02 ; 1.07) | **<0.001** |
| BGA: pH |  |  |  |  | 0.06 (0.03 ; 0.10) | **<0.001** |
| ^1^Hazard ratio (95% confidence interval) from multivariable Cox proportional hazards regression modeling. | | | | | | |
| Abbreviations: CI = Confidence Interval, HR = Hazard Ratio | | | | | | |

**Supplemental Table 11**. Sequential multivariable Cox proportional hazards modeling for 90-day overall survival: <65y

|  | **Model #1** | | **Model #2** | | **Model #3** | |
| --- | --- | --- | --- | --- | --- | --- |
| **Characteristic** | **HR (95% CI)**^1^ | **p-value** | **HR (95% CI)**^1^ | **p-value** | **HR (95% CI)**^1^ | **p-value** |
| Parental status |  |  |  |  |  |  |
| Without children | — |  | — |  | — |  |
| With children | 0.91 (0.80 ; 1.05) | 0.19 | 0.84 (0.73 ; 0.96) | **0.011** | 0.83 (0.72 ; 0.96) | **0.009** |
| Period (COVID-19 wave) |  |  |  |  |  |  |
| 1 | — |  | — |  | — |  |
| 2 | 0.76 (0.63 ; 0.91) | **0.003** | 0.72 (0.60 ; 0.87) | **<0.001** | 0.74 (0.61 ; 0.90) | **0.002** |
| 3 | 0.77 (0.66 ; 0.90) | **0.001** | 0.78 (0.66 ; 0.91) | **0.001** | 0.75 (0.64 ; 0.88) | **<0.001** |
| 4 | 0.87 (0.68 ; 1.12) | 0.29 | 0.86 (0.67 ; 1.10) | 0.24 | 0.84 (0.65 ; 1.09) | 0.19 |
| 5 | 0.93 (0.78 ; 1.11) | 0.45 | 0.83 (0.70 ; 0.995) | **0.044** | 0.86 (0.72 ; 1.04) | 0.12 |
| Age, years | 1.07 (1.06 ; 1.08) | **<0.001** | 1.07 (1.06 ; 1.07) | **<0.001** | 1.06 (1.05 ; 1.06) | **<0.001** |
| Male sex | 1.52 (1.33 ; 1.73) | **<0.001** | 1.50 (1.31 ; 1.71) | **<0.001** | 1.34 (1.16 ; 1.55) | **<0.001** |
| Number of comorbidities |  |  | 0.94 (0.68 ; 1.30) | 0.69 | 0.993 (0.71 ; 1.39) | 0.97 |
| Diabetes |  |  | 1.02 (0.71 ; 1.47) | 0.90 | 0.80 (0.55 ; 1.17) | 0.25 |
| Obesity |  |  | 1.16 (0.81 ; 1.66) | 0.42 | 0.95 (0.66 ; 1.38) | 0.79 |
| Body mass index (BMI), kg/m² |  |  | 1.03 (1.02 ; 1.05) | **<0.001** | 1.03 (1.01 ; 1.04) | **<0.001** |
| Hypertension |  |  | 0.88 (0.61 ; 1.26) | 0.49 | 0.82 (0.57 ; 1.18) | 0.29 |
| Congestive heart failure |  |  | 1.23 (0.84 ; 1.80) | 0.28 | 1.001 (0.67 ; 1.49) | >0.99 |
| Myocardial infarction |  |  | 1.17 (0.73 ; 1.87) | 0.50 | 1.23 (0.77 ; 1.99) | 0.39 |
| Cardiac arrythmia |  |  | 1.13 (0.77 ; 1.64) | 0.54 | 1.08 (0.74 ; 1.58) | 0.68 |
| Valvular disease |  |  | 1.08 (0.68 ; 1.73) | 0.74 | 1.33 (0.82 ; 2.15) | 0.24 |
| Peripheral vascular disease |  |  | 0.89 (0.59 ; 1.36) | 0.60 | 0.92 (0.60 ; 1.42) | 0.72 |
| Coagulopathy |  |  | 1.63 (1.11 ; 2.39) | **0.012** | 1.24 (0.85 ; 1.82) | 0.26 |
| Rheumatoid arthritis/collagen vas |  |  | 1.05 (0.68 ; 1.61) | 0.84 | 1.09 (0.70 ; 1.71) | 0.69 |
| Organ transplant |  |  | 1.26 (0.85 ; 1.87) | 0.25 | 1.25 (0.84 ; 1.86) | 0.27 |
| Chronic pulmonary disease |  |  | 0.87 (0.60 ; 1.27) | 0.48 | 1.002 (0.68 ; 1.48) | >0.99 |
| Pulmonary circulation disease |  |  | 1.77 (1.22 ; 2.56) | **0.003** | 1.30 (0.89 ; 1.89) | 0.17 |
| Renal failure |  |  | 2.03 (1.39 ; 2.98) | **<0.001** | 1.33 (0.89 ; 1.98) | 0.16 |
| Liver disease |  |  | 1.70 (1.16 ; 2.48) | **0.006** | 1.32 (0.89 ; 1.96) | 0.16 |
| Cerebrovascular disease |  |  | 1.70 (1.14 ; 2.55) | **0.009** | 1.59 (1.06 ; 2.39) | **0.025** |
| Paralysis |  |  | 0.64 (0.42 ; 0.994) | **0.047** | 0.56 (0.36 ; 0.88) | **0.011** |
| Dementia |  |  | 0.89 (0.49 ; 1.61) | 0.69 | 1.04 (0.57 ; 1.91) | 0.90 |
| Other neurological disorders |  |  | 1.22 (0.82 ; 1.79) | 0.32 | 1.11 (0.74 ; 1.66) | 0.63 |
| Solid tumor w/out metastasis |  |  | 1.38 (0.93 ; 2.05) | 0.11 | 1.40 (0.93 ; 2.10) | 0.11 |
| Metastatic cancer |  |  | 2.66 (1.72 ; 4.10) | **<0.001** | 2.62 (1.67 ; 4.09) | **<0.001** |
| Lymphoma |  |  | 1.40 (0.88 ; 2.22) | 0.15 | 1.38 (0.86 ; 2.21) | 0.18 |
| Na, mmol/L |  |  |  |  | 0.998 (0.99 ; 1.01) | 0.74 |
| K, mmol/L |  |  |  |  | 1.10 (1.001 ; 1.20) | **0.049** |
| Hemoglobin, g/dL |  |  |  |  | 0.93 (0.90 ; 0.96) | **<0.001** |
| Leukocytes count, x 10^9 / L |  |  |  |  | 0.98 (0.94 ; 1.02) | 0.30 |
| Lymphocyte count, x 10^9 / L |  |  |  |  | 1.02 (0.96 ; 1.09) | 0.51 |
| Neutrophil count, x 10^9 / L |  |  |  |  | 1.04 (1.000 ; 1.09) | 0.051 |
| Eosinophil count, x 10^9 / L |  |  |  |  | 0.18 (0.07 ; 0.51) | **0.001** |
| Platelet count, x 10^9 / L |  |  |  |  | 0.998 (0.997 ; 0.999) | **<0.001** |
| Serum creatinine, µmol/L |  |  |  |  | 0.999 (0.999 ; 1.000) | **0.019** |
| Urea, mmol/L |  |  |  |  | 1.03 (1.02 ; 1.04) | **<0.001** |
| C reactive protein (mg/L) |  |  |  |  | 1.001 (1.000 ; 1.002) | **0.015** |
| Protides, g/L |  |  |  |  | 0.98 (0.97 ; 0.99) | **<0.001** |
| ALT, U/L |  |  |  |  | 0.998 (0.996 ; 0.999) | **<0.001** |
| Bilirubin total, µmol/L |  |  |  |  | 1.005 (1.003 ; 1.007) | **<0.001** |
| Partial thromboplastin time (PTT) |  |  |  |  | 1.15 (1.02 ; 1.30) | **0.025** |
| Prothrombin time (%) |  |  |  |  | 0.998 (0.994 ; 1.003) | 0.43 |
| Glycemia, mmol/L |  |  |  |  | 1.05 (1.03 ; 1.07) | **<0.001** |
| HCO3- serum, mmol/L |  |  |  |  | 0.94 (0.92 ; 0.96) | **<0.001** |
| Calcium, mmol/L |  |  |  |  | 0.50 (0.35 ; 0.72) | **<0.001** |
| Fibrinogen, g/L |  |  |  |  | 1.003 (0.96 ; 1.05) | 0.89 |
| Procalcitonin, µg/L |  |  |  |  | 0.98 (0.96 ; 0.998) | **0.031** |
| Troponin, ng/L |  |  |  |  | 1.000 (1.000 ; 1.000) | 0.71 |
| Lacticodeshydrogenase, U/L |  |  |  |  | 1.002 (1.002 ; 1.002) | **<0.001** |
| Creatine kinase, U/L |  |  |  |  | 1.000 (1.000 ; 1.000) | 0.17 |
| D-Dimer (µg/L) |  |  |  |  | 1.000 (1.000 ; 1.000) | **0.036** |
| BGA: Oxygen saturation, % |  |  |  |  | 0.99 (0.98 ; 0.996) | **0.004** |
| BGA: PaO2, mmHg |  |  |  |  | 1.001 (0.999 ; 1.002) | 0.32 |
| BGA: PaCO2, mmHg |  |  |  |  | 0.98 (0.98 ; 0.991) | **<0.001** |
| BGA: HCO3-, mmol/L |  |  |  |  | 1.09 (1.06 ; 1.12) | **<0.001** |
| BGA: lactate, mmol/L |  |  |  |  | 0.998 (0.95 ; 1.05) | 0.95 |
| BGA: pH |  |  |  |  | 0.04 (0.01 ; 0.12) | **<0.001** |
| ^1^Hazard ratio (95% confidence interval) from multivariable Cox proportional hazards regression modeling. | | | | | | |
| Abbreviations: CI = Confidence Interval, HR = Hazard Ratio | | | | | | |

**Supplemental Table 12.** Sequential multivariable Cox proportional hazards modeling for 90-day overall survival: >65y

|  | **Model #1** | | **Model #2** | | **Model #3** | |
| --- | --- | --- | --- | --- | --- | --- |
| **Characteristic** | **HR (95% CI)**^1^ | **p-value** | **HR (95% CI)**^1^ | **p-value** | **HR (95% CI)**^1^ | **p-value** |
| Parental status |  |  |  |  |  |  |
| Without children | — |  | — |  | — |  |
| With children | 1.05 (0.97 ; 1.13) | 0.24 | 1.04 (0.97 ; 1.12) | 0.30 | 1.07 (0.99 ; 1.15) | 0.095 |
| Period (COVID-19 wave) |  |  |  |  |  |  |
| 1 | — |  | — |  | — |  |
| 2 | 0.75 (0.69 ; 0.82) | **<0.001** | 0.74 (0.68 ; 0.81) | **<0.001** | 0.78 (0.72 ; 0.85) | **<0.001** |
| 3 | 0.88 (0.82 ; 0.95) | **0.001** | 0.86 (0.80 ; 0.93) | **<0.001** | 0.86 (0.80 ; 0.93) | **<0.001** |
| 4 | 0.85 (0.74 ; 0.98) | **0.024** | 0.82 (0.71 ; 0.94) | **0.005** | 0.82 (0.71 ; 0.94) | **0.006** |
| 5 | 0.71 (0.65 ; 0.77) | **<0.001** | 0.68 (0.62 ; 0.74) | **<0.001** | 0.73 (0.67 ; 0.80) | **<0.001** |
| Age, years | 1.04 (1.03 ; 1.04) | **<0.001** | 1.05 (1.04 ; 1.05) | **<0.001** | 1.05 (1.05 ; 1.06) | **<0.001** |
| Male sex | 1.54 (1.45 ; 1.64) | **<0.001** | 1.52 (1.43 ; 1.62) | **<0.001** | 1.25 (1.17 ; 1.34) | **<0.001** |
| Number of comorbidities |  |  | 0.78 (0.53 ; 1.16) | 0.22 | 0.74 (0.50 ; 1.10) | 0.13 |
| Diabetes |  |  | 1.23 (0.82 ; 1.83) | 0.32 | 1.15 (0.77 ; 1.73) | 0.49 |
| Obesity |  |  | 1.20 (0.80 ; 1.81) | 0.38 | 1.28 (0.85 ; 1.92) | 0.24 |
| Body mass index (BMI), kg/m² |  |  | 1.03 (1.02 ; 1.04) | **<0.001** | 1.02 (1.008 ; 1.03) | **<0.001** |
| Hypertension |  |  | 1.03 (0.69 ; 1.54) | 0.87 | 1.15 (0.77 ; 1.72) | 0.49 |
| Congestive heart failure |  |  | 1.40 (0.94 ; 2.09) | 0.10 | 1.40 (0.93 ; 2.10) | 0.10 |
| Myocardial infarction |  |  | 1.24 (0.82 ; 1.89) | 0.31 | 1.32 (0.87 ; 2.01) | 0.19 |
| Cardiac arrythmia |  |  | 1.29 (0.86 ; 1.92) | 0.22 | 1.33 (0.89 ; 1.99) | 0.16 |
| Valvular disease |  |  | 1.20 (0.80 ; 1.82) | 0.38 | 1.27 (0.84 ; 1.92) | 0.26 |
| Peripheral vascular disease |  |  | 1.24 (0.82 ; 1.85) | 0.31 | 1.29 (0.86 ; 1.94) | 0.22 |
| Coagulopathy |  |  | 1.49 (0.99 ; 2.23) | 0.057 | 1.24 (0.82 ; 1.87) | 0.31 |
| Rheumatoid arthritis/collagen vas |  |  | 1.38 (0.90 ; 2.11) | 0.14 | 1.60 (1.04 ; 2.44) | **0.031** |
| Organ transplant |  |  | 2.08 (1.36 ; 3.20) | **<0.001** | 1.96 (1.28 ; 3.02) | **0.002** |
| Chronic pulmonary disease |  |  | 1.24 (0.83 ; 1.85) | 0.30 | 1.40 (0.94 ; 2.10) | 0.10 |
| Pulmonary circulation disease |  |  | 1.29 (0.86 ; 1.93) | 0.22 | 1.31 (0.87 ; 1.97) | 0.19 |
| Renal failure |  |  | 1.36 (0.91 ; 2.04) | 0.13 | 1.36 (0.91 ; 2.05) | 0.13 |
| Liver disease |  |  | 1.27 (0.84 ; 1.94) | 0.26 | 1.28 (0.84 ; 1.95) | 0.25 |
| Cerebrovascular disease |  |  | 1.07 (0.71 ; 1.61) | 0.74 | 1.19 (0.79 ; 1.79) | 0.41 |
| Paralysis |  |  | 1.28 (0.84 ; 1.94) | 0.25 | 1.41 (0.93 ; 2.14) | 0.11 |
| Dementia |  |  | 0.97 (0.65 ; 1.46) | 0.90 | 1.19 (0.79 ; 1.78) | 0.40 |
| Other neurological disorders |  |  | 1.38 (0.92 ; 2.07) | 0.12 | 1.64 (1.09 ; 2.46) | **0.017** |
| Solid tumor w/out metastasis |  |  | 1.14 (0.76 ; 1.72) | 0.52 | 1.28 (0.85 ; 1.93) | 0.23 |
| Metastatic cancer |  |  | 2.06 (1.36 ; 3.13) | **<0.001** | 2.30 (1.51 ; 3.49) | **<0.001** |
| Lymphoma |  |  | 1.57 (1.03 ; 2.39) | **0.037** | 1.76 (1.16 ; 2.69) | **0.008** |
| Na, mmol/L |  |  |  |  | 1.02 (1.010 ; 1.02) | **<0.001** |
| K, mmol/L |  |  |  |  | 1.08 (1.03 ; 1.13) | **0.003** |
| Hemoglobin, g/dL |  |  |  |  | 0.99 (0.97 ; 1.005) | 0.17 |
| Leukocytes count, x 10^9 / L |  |  |  |  | 0.99 (0.97 ; 1.003) | 0.10 |
| Lymphocyte count, x 10^9 / L |  |  |  |  | 1.01 (0.99 ; 1.04) | 0.33 |
| Neutrophil count, x 10^9 / L |  |  |  |  | 1.02 (1.004 ; 1.04) | **0.016** |
| Eosinophil count, x 10^9 / L |  |  |  |  | 0.19 (0.11 ; 0.33) | **<0.001** |
| Platelet count, x 10^9 / L |  |  |  |  | 0.998 (0.998 ; 0.999) | **<0.001** |
| Serum creatinine, µmol/L |  |  |  |  | 0.999 (0.999 ; 1.000) | **<0.001** |
| Urea, mmol/L |  |  |  |  | 1.02 (1.01 ; 1.03) | **<0.001** |
| C reactive protein (mg/L) |  |  |  |  | 1.003 (1.003 ; 1.004) | **<0.001** |
| Protides, g/L |  |  |  |  | 0.992 (0.99 ; 0.996) | **<0.001** |
| ALT, U/L |  |  |  |  | 0.998 (0.997 ; 0.998) | **<0.001** |
| Bilirubin total, µmol/L |  |  |  |  | 1.003 (1.001 ; 1.005) | **0.006** |
| Partial thromboplastin time (PTT) |  |  |  |  | 1.17 (1.08 ; 1.26) | **<0.001** |
| Prothrombin time (%) |  |  |  |  | 0.997 (0.995 ; 0.999) | **0.003** |
| Glycemia, mmol/L |  |  |  |  | 1.04 (1.03 ; 1.05) | **<0.001** |
| HCO3- serum, mmol/L |  |  |  |  | 0.97 (0.96 ; 0.98) | **<0.001** |
| Calcium, mmol/L |  |  |  |  | 0.54 (0.44 ; 0.68) | **<0.001** |
| Fibrinogen, g/L |  |  |  |  | 0.92 (0.89 ; 0.94) | **<0.001** |
| Procalcitonin, µg/L |  |  |  |  | 0.996 (0.99 ; 1.006) | 0.41 |
| Troponin, ng/L |  |  |  |  | 1.000 (1.000 ; 1.000) | **<0.001** |
| Lacticodeshydrogenase, U/L |  |  |  |  | 1.002 (1.002 ; 1.002) | **<0.001** |
| Creatine kinase, U/L |  |  |  |  | 1.000 (1.000 ; 1.000) | **0.006** |
| D-Dimer (µg/L) |  |  |  |  | 1.000 (1.000 ; 1.000) | **0.004** |
| BGA: Oxygen saturation, % |  |  |  |  | 0.99 (0.99 ; 0.994) | **<0.001** |
| BGA: PaO2, mmHg |  |  |  |  | 0.999 (0.998 ; 1.000) | **0.022** |
| BGA: PaCO2, mmHg |  |  |  |  | 0.997 (0.993 ; 1.001) | 0.17 |
| BGA: HCO3-, mmol/L |  |  |  |  | 1.03 (1.02 ; 1.05) | **<0.001** |
| BGA: lactate, mmol/L |  |  |  |  | 1.06 (1.03 ; 1.09) | **<0.001** |
| BGA: pH |  |  |  |  | 0.06 (0.03 ; 0.13) | **<0.001** |
| ^1^Hazard ratio (95% confidence interval) from multivariable Cox proportional hazards regression modeling. | | | | | | |
| Abbreviations: CI = Confidence Interval, HR = Hazard Ratio | | | | | | |

**** **Supplemental Figure 1. Distribution of all hospitalized patients by COVID-19 wave over the whole study period (waves 1 to 5)**

**Supplemental Figure 2. Day-90 overall survival by parental status over the whole study period**

**Supplemental Figure 3. Day-90 overall survival by parental status over the 1st COVID-19 wave**

**Supplemental Figure 4. Day-90 overall survival by parental status over the 2nd COVID-19 wave**

**Supplemental Figure 5. Day-90 overall survival by parental status over the 3rd COVID-19 wave**

**Supplemental Figure 6. Day-90 overall survival by parental status over the 4th COVID-19 wave**

**Supplemental Figure 7. Day-90 overall survival by parental status over the 5th COVID-19 wave**

Supplemental Figure 8. Parental status association with 90-day overall survival according to age (linear)

Cox proportional hazards with interaction between parental status (binary variable) and age (continuous variable modeled linearly)


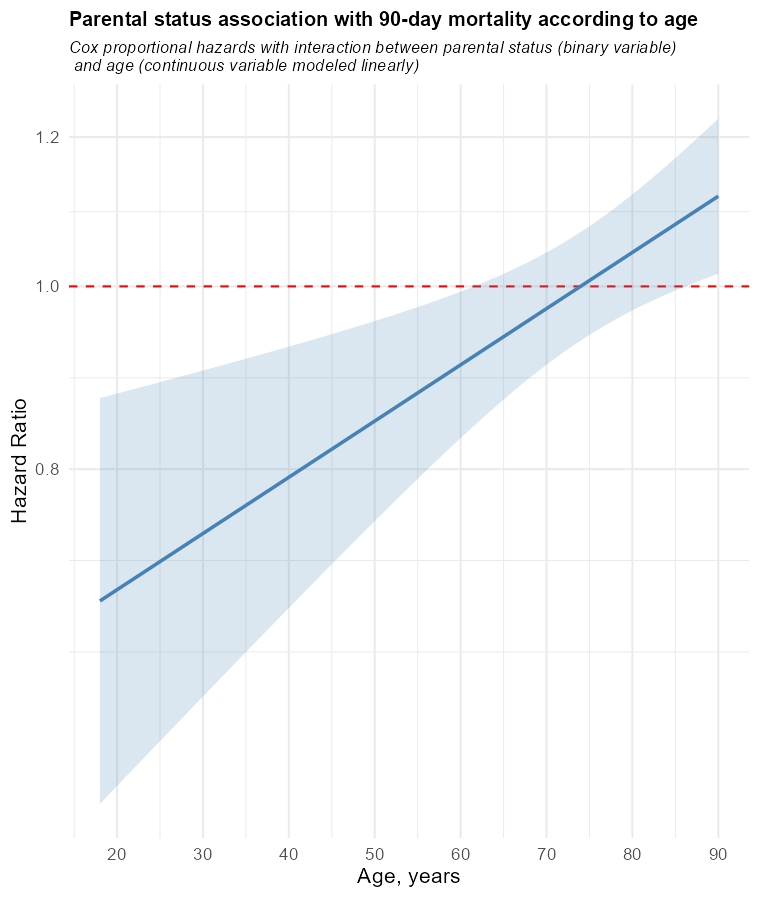


Supplemental Figure 9. Parental status association with 90-day overall survival according to age (5-knots restricted cubic spline)


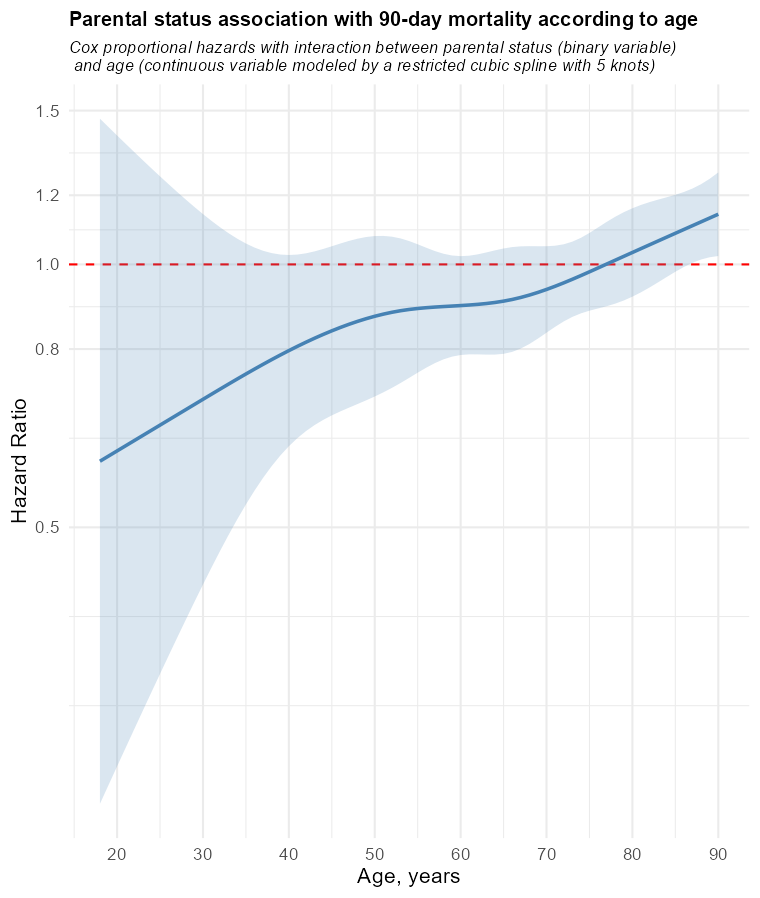
Cox proportional hazards with interaction between parental status (binary variable) and age (continuous variable modeled as a restricted cubicspline with 5 knots)


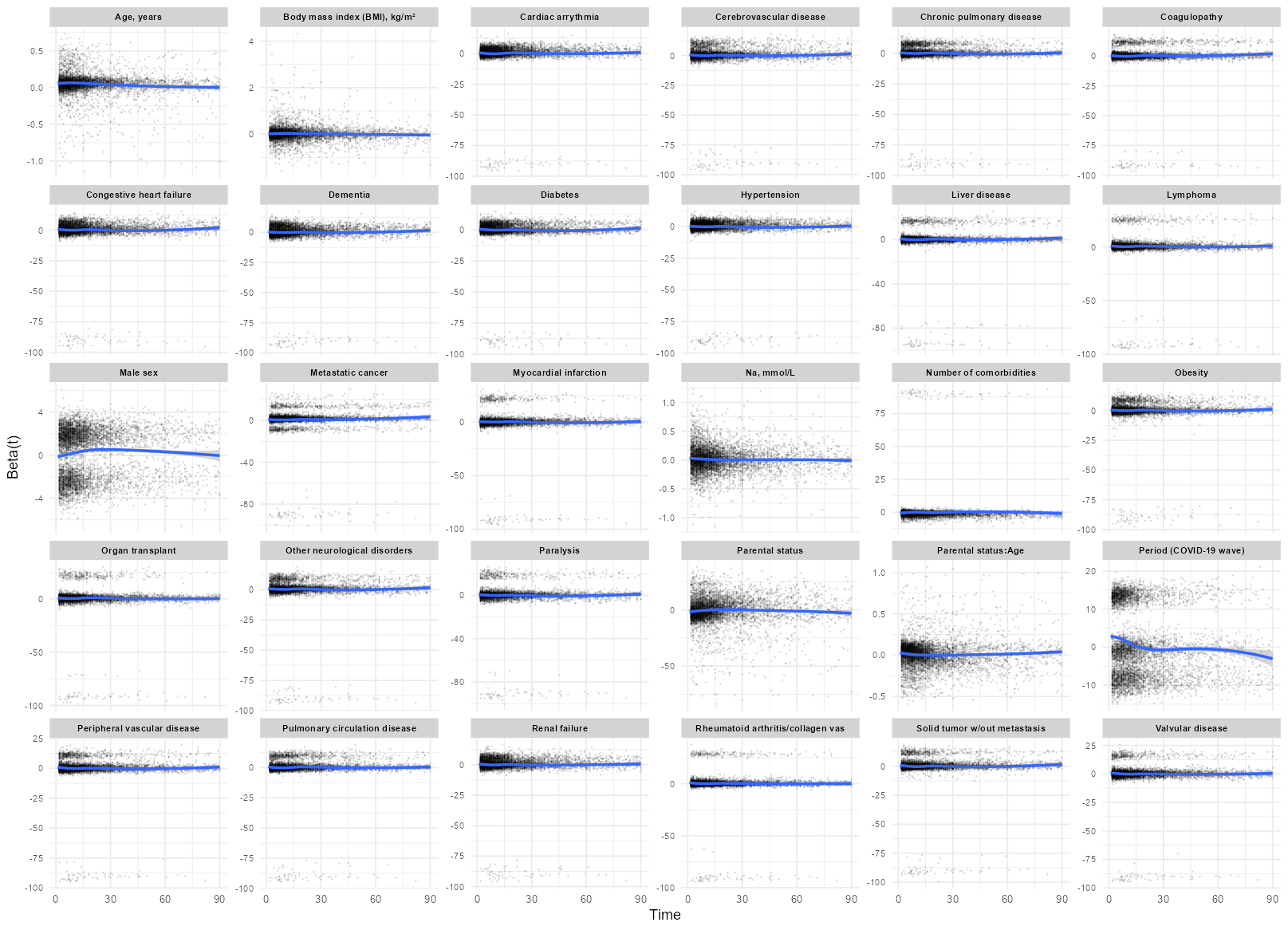
Supplemental Figure 10. Scaled Schoenfeld residuals, part 1

**
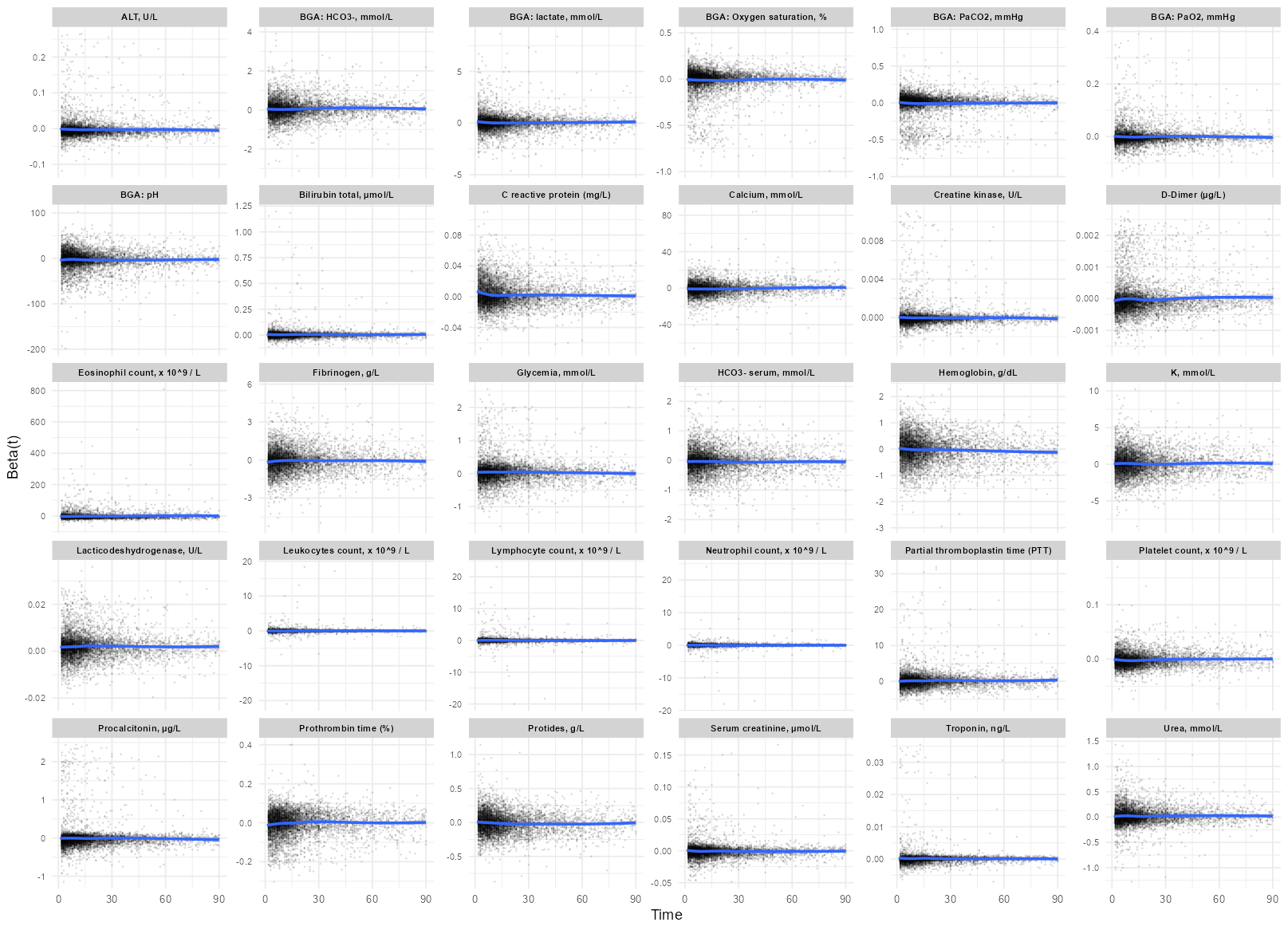
Supplemental Figure 11.** Scaled Schoenfeld residuals, part 2
